# The transcription factor IRF8 drives tumor-specific exhaustion in CD8^+^ T cells

**DOI:** 10.1084/jem.20252115

**Published:** 2026-06-22

**Authors:** Marco Ongaro, Romane Thouenon, Isaac Crespo, Alexandre Dumez, Mélanie Charmoy, Eduardo Roman Camacho, Victoria J. Morel, Daniela Cropp, Emma Desponds, Giulio Zanette, Yi-Chuan Wang, Letizia Vergili, Laure Tillé, Inés Di Resta, Marine M. Leblond, Jésus Corria Osorio, Ping-Chih Ho, Werner Held, Grégory Verdeil

**Affiliations:** 1 Department of Fundamental Oncology UNIL, University of Lausanne, Lausanne, Switzerland; 2 Ludwig Institute for Cancer Research, University of Lausanne, Lausanne, Switzerland; 3 SIB Swiss Institute of Bioinformatics, Lausanne, Switzerland

## Abstract

T cell exhaustion is a major obstacle to effective immunotherapy in cancer and chronic infection. Here, we identify the transcription factor IRF8 as a tumor-specific regulator of CD8^+^ T cell exhaustion. IRF8 is strongly expressed in tumor-reactive CD8^+^ T cells but not during chronic viral infection. Its expression is induced by TCR signaling and can be suppressed by type I IFN (IFN-I). Sustained IFN-I signaling, a hallmark of chronic infection, correlates with reduced chromatin accessibility at the *Irf8* locus and progressive repression of *Irf8* expression. In tumor-specific CD8^+^ T cells, IRF8 overexpression enhanced TOX expression while reducing IFNγ, granzyme B, and TNF production. Conversely, *Irf8* deficiency diminished exhaustion, restored effector functions, and improved tumor control. Mechanistically, IRF8 directly binds the *Tox* locus and promotes its transcription. We further show that additional IRF-family transcription factors contribute similarly to the exhausted T cell program, identifying this transcriptional network as a key regulator of tumor-associated T cell dysfunction.

## Teaser

The study by Ongaro et al. identifies IRF8 as a tumor-specific regulator driving CD8^+^ T cell exhaustion: induced by TCR but suppressed by chronic IFN-I, IRF8 promotes TOX and represses effector functions; targeting IRF8/IRF-family network restores anti-tumor immunity, informing immunotherapy strategies for cancer therapy.

## Introduction

In chronic infection and cancer, persistent antigen exposure drives CD8^+^ T cells toward exhaustion, a dysfunctional differentiation state characterized by progressive loss of effector function, expression of multiple inhibitory receptor, and decreased proliferative capacity (Wherry, 2011). Recent advances have provided insights into the complex transcriptional networks regulating exhaustion in chronic infection and in cancer (Chen et al., 2026; Doering et al., 2012; Jansen et al., 2019; Philip et al., 2017; Sun and Dong, 2026; Wherry and Kurachi, 2015; Wu et al., 2026), with evidence for substantial differences between the two conditions due to their distinct microenvironments (Luxenburger et al., 2026; McLane et al., 2019; Philip and Schietinger, 2019; Tille et al., 2023). Among the central regulators of this state, several families of transcription factors (TFs) play a dominant function, including TOX (Alfei et al., 2019; Khan et al., 2019; Scott et al., 2019), NFAT (Martinez et al., 2015; Tille et al., 2023), and NR4A (Chen et al., 2019) families. Several members of the IFN regulatory factor (IRF) family have also been associated with CD8^+^ T cell exhaustion, including IRF2, IRF4, IRF5, and IRF7 (Kasmani et al., 2023; Lukhele et al., 2022; Mai et al., 2025; Man et al., 2017; Seo et al., 2021), raising interest in their role in CD8^+^ T cell differentiation fates. Through analysis of the inferred TF activity based on the analysis of regulatory programs of CD8^+^ T cells from multiple cancer and chronic infection studies, we identified IRF8 among the most active TFs in exhausted CD8^+^ T cells in a tumor-specific context. Despite studies showing its crucial role in myeloid cells (Aliberti et al., 2003; Grajales-Reyes et al., 2015; Kurotaki et al., 2014; Yanez et al., 2015), B cells (Carotta et al., 2014) and subsets of CD4^+^ T cells (Humblin et al., 2017; Ouyang et al., 2011), the role of IRF8 in CD8^+^ T cells is not fully understood, as different studies reached divergent conclusions. IRF8 was reported to drive effector differentiation of CD8^+^ T cells in a mouse model of graft-versus-host disease (Miyagawa et al., 2012) but also to restrain excessive CD8^+^ T cells proliferation during herpes simplex virus 1 infection (Sun et al., 2016). More recently, IRF8 was found to regulate the 3D chromatin architecture by linking promoters to distal enhancer regions, thereby improving gene transcription through interaction with the TF CTCF in exhausted CD8^+^ T cells. Based on the known function of IRF4 (Li et al., 2025), the close structural homology of IRF8 based on phylogenetic analysis of the IRF family (Antonczyk et al., 2019) raises the possibility that IRF8 plays a role in the regulation of differentiation of CD8^+^ T cells. Indeed, the two TFs share several binding partners, such as the AP-1 factors JUNB and BATF, PU.1, ETV6, and other IRFs, pointing toward similar and possibly redundant regulatory mechanisms for certain IRF family members.

In this study, we characterized the function of IRF8 in the regulation of CD8^+^ T cell exhaustion in tumors and chronic viral infection. We found that IRF8 expression is high in murine and human CD8^+^ tumor-infiltrating lymphocytes (TILs) and is driven by TCR in the tumor microenvironment (TME) but is repressed during chronic infection. The *Irf8* locus was less accessible and less expressed in virus-specific CD8^+^ T cells from lymphocytic choriomeningitis virus clone 13 (LCMV cl13) infected animals compared with tumor-specific CD8^+^ T cells. Type I IFN, typically associated with chronic infections, can diminish IRF8 expression in CD8^+^ T cells. IRF8 KO in CD8^+^ T cells improved the anti-tumor response by dampening TOX expression and increasing the production of effector molecules, while IRF8 overexpression (OE) aggravated their exhausted phenotype. By comparing the phenotype and transcriptome of IRF8 KO with IRF2 and IRF4 KO CD8^+^ TILs, we showed convergence of the IRF family for the regulation of TOX-dependent T cell exhaustion. Altogether, we highlighted the role of IRF8 as a novel TF regulating tumor-specific CD8^+^ T cell exhaustion, which could unravel new therapeutic opportunities to better harness immunotherapies.

## Results

### IRF8 is highly expressed in exhausted CD8^+^ TILs in mouse and human cancer

**Figure 1.**
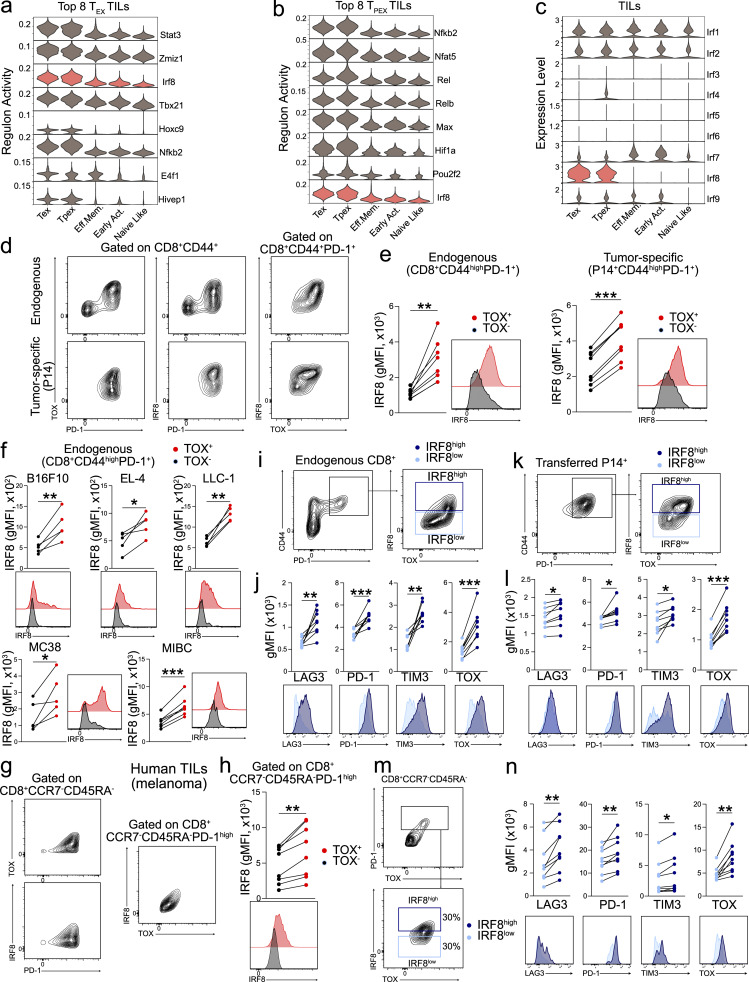
**IRF8 is highly expressed in exhausted CD8** ^**+**^ **TILs in murine and human cancer. (a and b)** Top eight discriminant regulon signature in T_EX_ (a) and T_PEX_ (b) CD8^+^ TILs based on publicly available datasets as described in the Materials and methods section. **(c)** IRF TF family members gene expression levels in CD8 TILs based on publicly available scRNA-seq datasets for different cancer studies, as described in the Materials and methods section. **(d)** Representative FACS plots showing TOX, PD-1, and IRF8 expression in endogenous and tumor-specific (P14) CD8^+^ TILs in indicated populations 14 days after B16-gp33 melanoma engraftment in C57/BL6 mice. **(e)** Quantification of IRF8 protein levels in TOX^−^ and TOX^+^ CD8^+^ TILs among endogenous (left) and transferred tumor-specific P14 (right) CD8^+^CD44^high^PD-1^+^ with representative FACS histogram (TOX^+^ in red, TOX^−^ in black). *n* = 7, representative data from three independent experiments. **(f)** Flow cytometric analysis of IRF8 expression levels comparing TOX^+^ and TOX^−^ CD8^+^CD44^high^PD-1^+^ endogenous TILs population in B16F10 melanoma (*n* = 5), EL-4 thymoma (*n* = 5), LLC1 (*n* = 5), murine colon adenocarcinoma (MC38, *n* = 5), and orthotopic inducible MIBC (*n* = 7) with representative histograms. **(g)** Representative FACS plots showing TOX, PD-1, and IRF8 expression in human melanoma metastasis samples in indicated populations. IRF8 protein levels in TOX^−^ and TOX^+^ CD8 TILs (among CD8^+^CCR7^−^CD45RA^−^PD-1^high^) from nine human melanoma metastasis samples with representative FACS histogram (TOX^+^ in red, TOX^−^ in black). *n* = 9, data pooled from two independent experiments. **(h, i, k, and m)** Representative FACS plots showing gating strategy for IRF8^high^ and IRF8^low^ mouse CD8^+^ TILs in endogenous (i) and P14 (k) cells from experiment in d and e, and human CD8^+^ TILs cells from experiment in g, h, and m. **(j, l, and n)** Quantification of LAG3, PD-1, TIM3, and TOX protein levels comparing IRF8^high^ versus IRF8^low^ cells in the respective populations indicated above. **(e, f, h, j, l, and n)** Statistical analysis was done by paired two-tailed Student’s *t* test. *P < 0.05, **P < 0.01, and ***P < 0.001.

To identify TFs specifically active in exhausted CD8^+^ T cells, we inferred regulatory programs (regulons) from publicly available single-cell RNA sequencing (scRNA-seq) profiles from mouse CD8^+^ TILs using the SCENIC workflow (Aibar et al., 2017). We found that the regulon signature of IRF8 was among the top discriminant regulons for both progenitor exhausted (T_PEX_) and terminally exhausted (T_EX_) CD8^+^ T cells (Fig. 1, a and b). By analyzing mRNA expression levels from the same datasets, we observed a strong and specific expression of IRF8 in both T_PEX_ and T_EX_ compared with other IRF family members (Fig. 1 c), supporting a role for IRF8 in regulating CD8^+^ T cell exhaustion. To assess IRF8 protein expression in exhausted CD8^+^ T cells, we engrafted B16-gp33 melanoma cells by s.c. injection into C57/BL6 mice (B6), transferred with TCR transgenic CD8^+^ T cells that recognize the LCMV-derived gp_33–41_ (gp33) epitope (P14 cells) and analyzed tumors after 14 days after tumor engraftment. TOX^+^ CD44^high^PD-1^+^ CD8^+^ T cells showed significantly increased IRF8 levels compared with TOX^−^ CD44^high^PD-1^+^ CD8^+^ T cells in the same tumor in both endogenous (host) CD8^+^ and tumor-specific P14 compartments, indicating that IRF8 is specifically expressed in exhausted CD8^+^ T cells (Fig. 1, d and e). We confirmed these results in B16F10 melanoma, murine EL-4 thymoma, LLC1, colon adenocarcinoma (MC38), and in an inducible orthotopic muscle invasive bladder cancer model (MIBC) (Leblond et al., 2020) (Fig. 1 f). We confirmed IRF8 OE in human exhausted CD8^+^ TILs, analyzing tumor samples from nine metastatic melanoma patients. IRF8 expression was significantly higher in TOX^+^PD-1^high^ compared with TOX^−^PD-1^high^ CD8^+^ T cells from the same tumor (Fig. 1, g and h), indicating that IRF8 expression is not restricted to murine CD8^+^ T cells. IRF8^high^ CD8^+^ T cells showed higher expression of exhaustion associated molecules, such as LAG3, PD-1, TIM3, and TOX, compared with IRF8^low^ CD8^+^ T cells, showing that IRF8 levels correlate with the exhaustion state in both murine and human TILs (Fig. 1, i–n). Altogether these results suggest that IRF8 is associated with CD8^+^ T cell exhaustion in cancer.

### Exhausted CD8^+^ T cells specifically express high level of IRF8 in cancer but not during chronic LCMV cl13 infection

**Figure 2.**
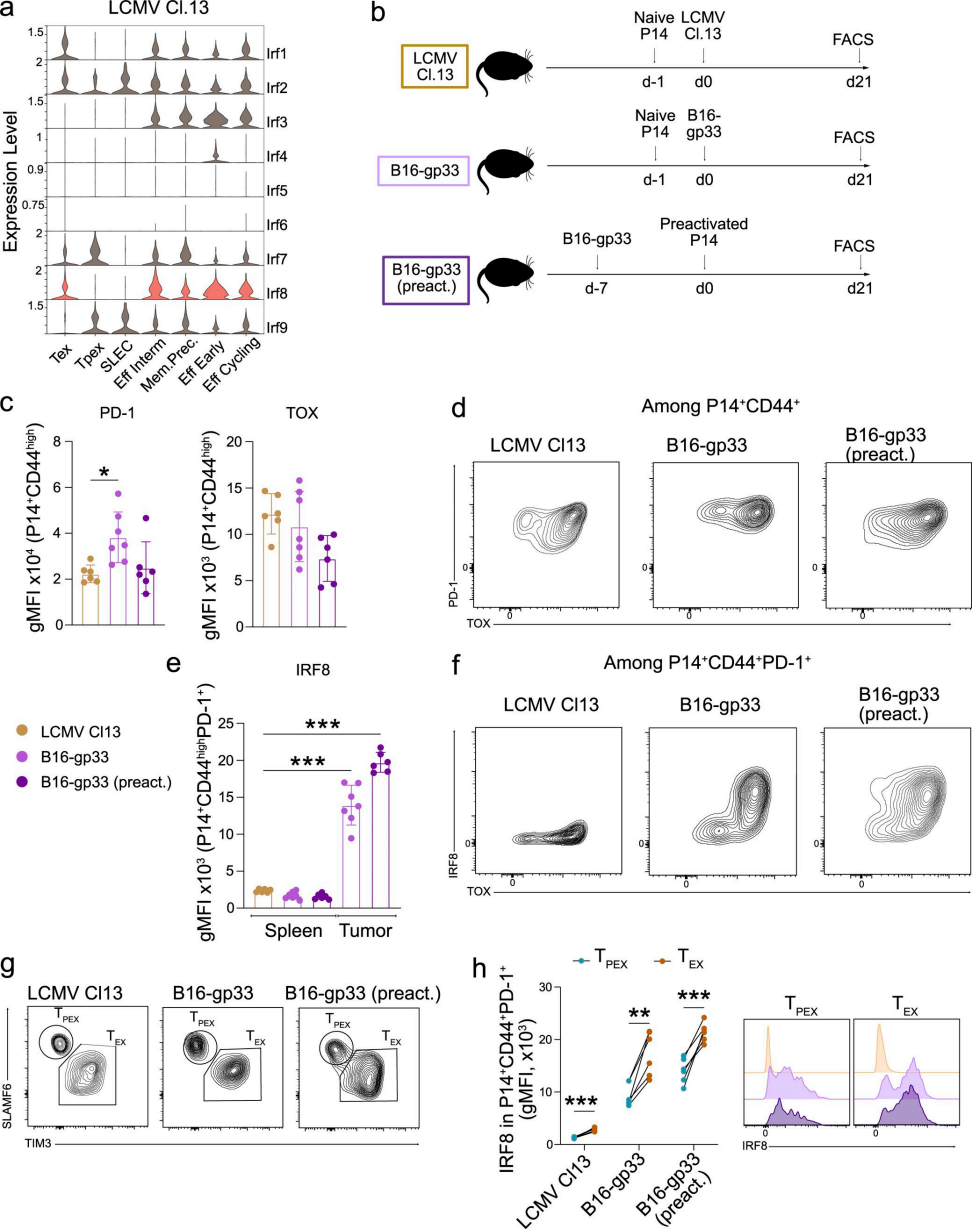
**Exhausted CD8** ^**+**^ **T cells specifically express IRF8 in cancer. (a)** Expression of IRF TF family members in virus-specific CD8 T cells based on publicly available scRNA-seq datasets from different LCMV infection studies (Material and methods). **(b)** Schematic representation of experimental procedure used to compare IRF8 levels in tumor- versus virus-specific exhausted CD8^+^ T cells. In the LCMV Cl13 chronic infection (top), 10^3^ naive P14 CD8^+^ T cells have been transferred into Vβ5 mice 1 day prior to infection with LCMV Cl13. In the B16-gp33 condition (middle), 2 × 10^6^ naive P14 CD8^+^ T cells have been transferred 1 day prior to B16-gp33 tumor engraftment (0.5 × 10^5^ cells s.c.) in B6 mice. In the B16-gp33 (preact.) condition (bottom), B16-gp33 tumors where engrafted in B6 mice 7 days prior to adoptive transfer of 10^6^ P14 CD8^+^ T cells preactivated *in vitro* during 48 h. Readout was done 21 days after *in vivo* antigen encounter. **(c)** Flow cytometric quantification of PD-1 (left) and TOX (right) in the indicated conditions. **(d)** Representative FACS plots for c showing TOX and PD-1 expression in the indicated population. **(e)** IRF8 protein quantification in transferred P14 CD8^+^CD44^+^PD-1^+^ CD8^+^ T cells in spleens from the three conditions in b and tumors from B16-gp33 and B16-gp33 (preact.). **(f)** Representative FACS plots for c showing TOX and IRF8 expression in the indicated population. **(g)** Representative FACS plots showing gating strategy for SLAMF6^+^TIM3^−^ T_PEX_ and SLAMF6^−^TIM3^+^ T_EX_ among P14 CD8^+^CD44^high^PD-1^+^ cells in indicated populations. **(h)** IRF8 quantification in T_PEX_ and T_EX_ in the indicated condition (left) and representative FACS histograms (right). In b–h, pooled data are from two independent experiments with *n* = 6 for LCMV Cl13, *n* = 7 for B16-gp33, and *n* = 6 for B16-gp33 (preact.). In c, statistical analysis was done with ordinary two-way ANOVA. In e, statistical analysis was done with ordinary two-way ANOVA comparing each column with the LCMV Cl13 condition. In h, statistical analysis was done by multiple paired two-tailed Student’s *t* tests. Data in c and e are represented as mean with SD. *P < 0.05, **P < 0.01, and ***P < 0.001.

**Figure S1.**
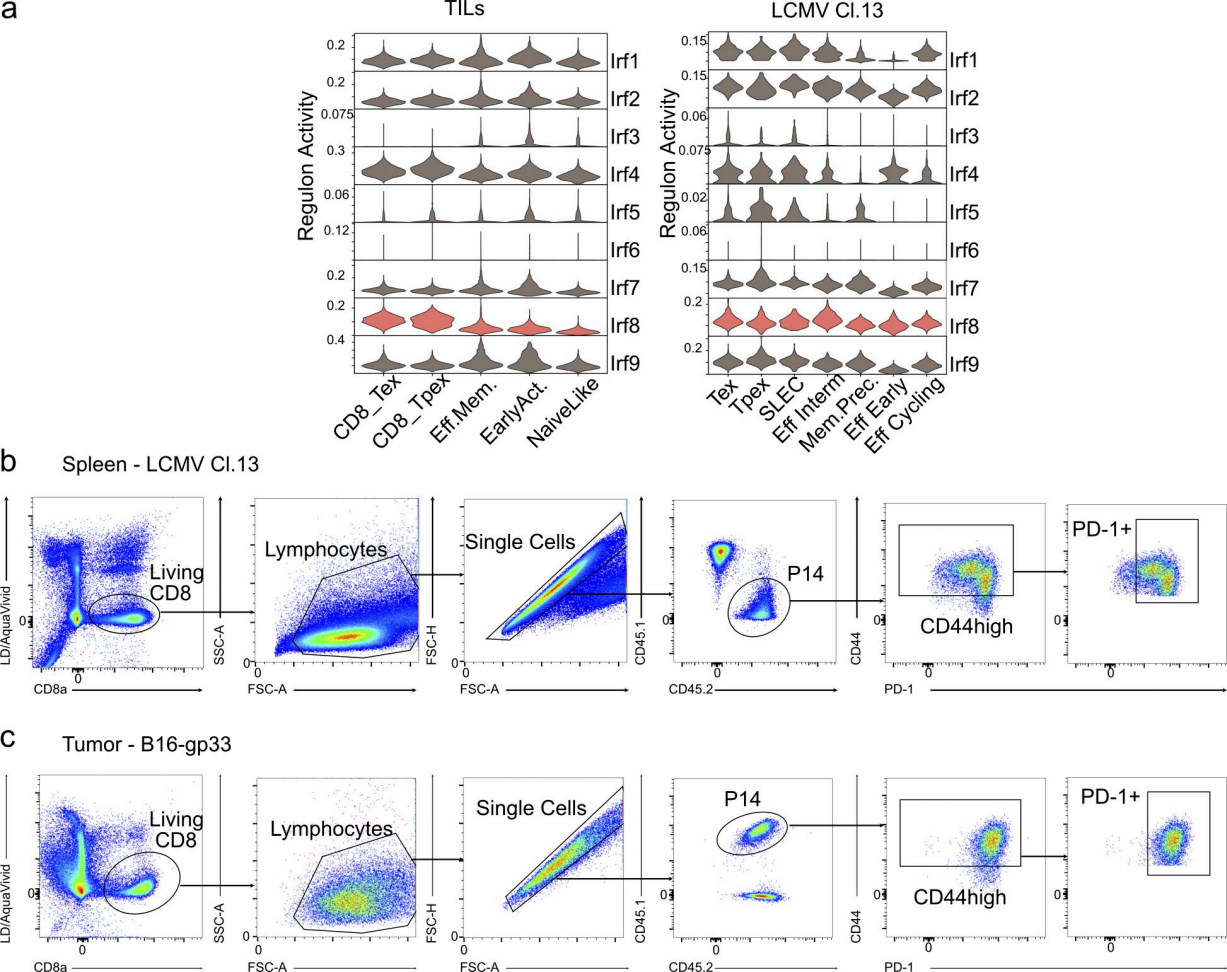
**Comparison of virus- and tumor-specific CD8** ^**+**^ **T cells. (a)** Regulon signature of IRF family members in different cell states of CD8 TILs (left) and virus-specific CD8 T cells (right) based on publicly available datasets as described in the Materials and methods section. **(b and c)** Representative gating strategy used to assess IRF8 protein levels in P14 CD8^+^ T cells in spleen of LCMV Cl13-infected Vβ5 mice (b) and in B16-gp33 tumors (c) as described in Fig. 2 b.

Compared with the expression of IRF8 in exhausted CD8^+^ TILs, scRNA-seq data showed that *Irf8* mRNA levels were lower in exhausted CD8^+^ T cells responding to chronic LCMV cl13 infection in both T_EX_ and T_PEX_ (Fig. 2 a). Moreover, inferred IRF8 regulon activity was not specific to T_PEX_ and T_EX_ in this setting (Fig. S1 a). To compare IRF8 expression at the protein level between cancer and chronic infection, we adoptively transferred naive P14 cells into B6 mice 1 day prior to s.c. injection with B16-gp33 tumor cells. In parallel, we transferred naive P14 cells into TCRβ (Vβ5) transgenic mice 1 day prior to the infection with LCMV cl13, which causes chronic infection. Vβ5 mice display a reduced endogenous T cell response to LCMV and, similarly to CD4-depleted B6, have higher LCMV cl13 titers compared with B6 mice (Utzschneider et al., 2016; Utzschneider et al., 2013). 3 wk after tumor grafting or chronic infection, P14 cells (Fig. 2 b) displayed equivalent levels of TOX but expressed slightly higher PD-1 levels in the tumor compared with chronic infection, indicating the establishment of exhaustion across all conditions (Fig. 2, c and d). IRF8 was highly expressed in tumor-derived CD44^high^ PD-1^+^ P14 cells but was expressed at low levels in the corresponding chronic infection–derived P14 cells present in the spleen in both T_EX_ and T_PEX_ (Fig. 2, e–h; and Fig. S1, b and c). IRF8 was comparably low in spleens of tumor-bearing mice, confirming that IRF8 OE was restricted to tumor-associated CD8^+^ T cells (Fig. 2, e and f).

We also compared IRF8 expression in the naive P14 cells responding to tumors with that of *in vitro* preactivated P14 cells that were transferred into B6 mice 7 days after tumor engraftment (Fig. 2, b–h). The data confirmed our previous results, indicating that tumor-specific CD8^+^ TILs express IRF8 independently of whether priming occurred *in vivo* or *in vitro*. We further observed that IRF8 expression was significantly higher in tumor-derived SLAMF6^−^ TIM3^+^ T_EX_ compared with SLAMF6^+^ TIM3^−^ T_PEX_ cells, in line with *Irf8* mRNA expression (Fig. 1 c). IRF8 was also higher in chronic infection–derived T_EX_ than T_PEX_ cells, although the overall expression was much lower than in tumors. Altogether, these observations suggest that IRF8 OE is associated with CD8^+^ T cell exhaustion specifically in the context of cancer.

### TCR stimulation drives IRF8 expression in the TME

**Figure 3.**
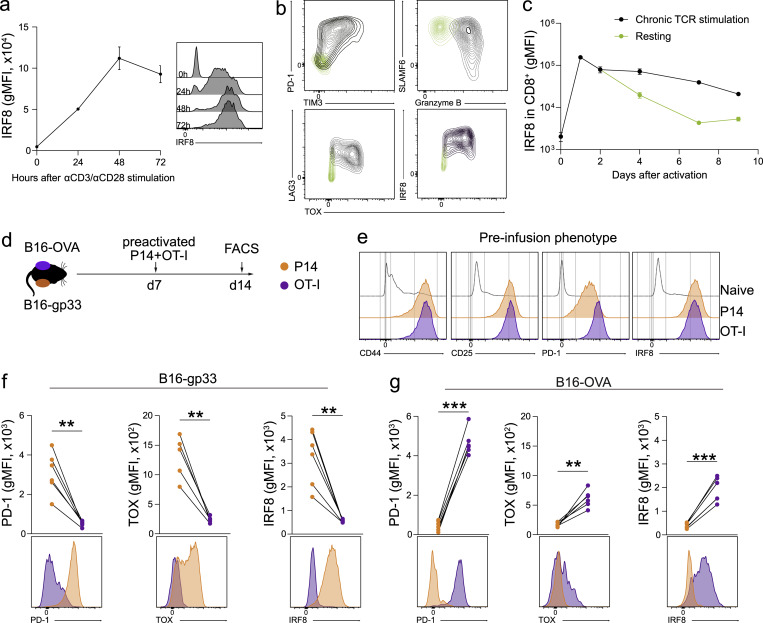
**TCR stimulation drives IRF8 expression in the TME. (a)** Kinetics of IRF8 protein levels of enriched CD8 T cells activated with plate-bound αCD3 and soluble αCD28 antibodies with representative histogram (*n* = 3 biological replicates, representative data from two independent experiments). **(b)** Representative FACS plots showing PD-1, TIM3, SLAMF6, GZMB, LAG3, TOX, and IRF8 expression in resting (green) and *in vitro* chronically TCR-stimulated CD8^+^ T cells (black) as described in the Materials and methods. **(c)** IRF8 quantification in CD8^+^ T cells from experiment in b. *n* = 3 biological replicates, data representative of two independent experiments. **(d)** Schematic representation of experimental procedure used to address TCR dependency of IRF8 expression *in vivo* in the TME. B6 mice were engrafted with B16-gp33 and B16-OVA on opposite flanks. 10^6^ preactivated P14 and 10^6^ preactivated OT-I T cells were co-transferred at day 7 after tumor initiation. Readout was performed on day 14 after tumor engraftment. **(e)** FACS histograms showing expression of CD44, CD25, PD-1, and IRF8 on the day of ACT in naive (black dotted line), P14 (orange), and OT-I (purple) CD8^+^ T cells after 48 h of *in vitro* activation with gp33 or OVA peptide. **(f and g)** Flow cytometric analysis of PD-1, TOX, and IRF8 protein levels in P14^+^ and OT-I^+^ CD8 T cells recovered from B16-gp33 (f) or B16-OVA (g) tumors 7 days after co-transfer as described in d. *n* = 6, representative data from two independent experiments. In f and g, statistical analysis was done by paired two-tailed Student’s *t* test. **P < 0.01 and ***P < 0.001.

**Figure S2.**
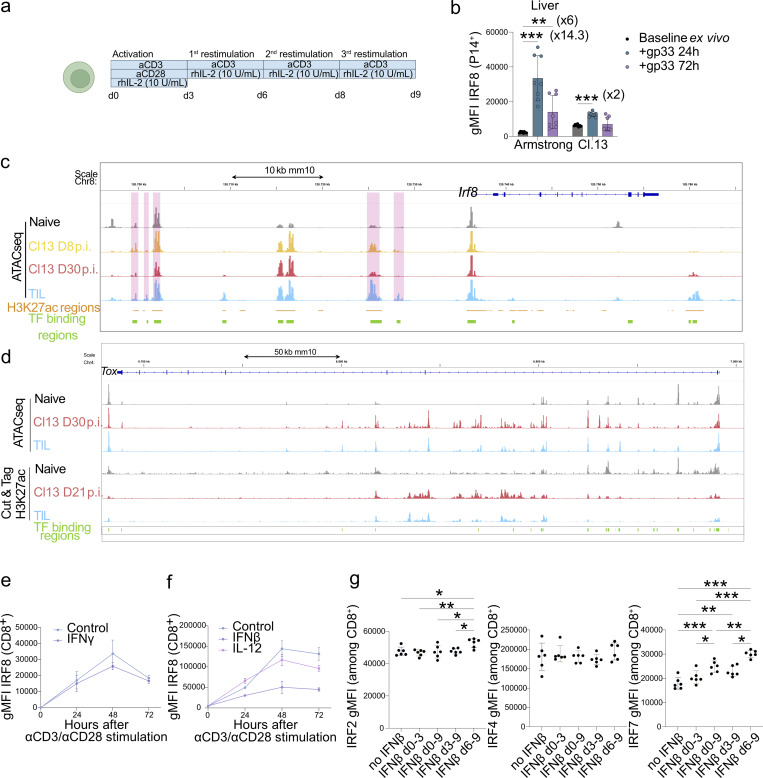
**Regulation of IRF8 expression in CD8 T cells is dependent on TCR stimulation and is repressed during chronic LCMV Cl13 infection. (a)** Experimental setup of the chronic TCR stimulation *in vitro* used to assess IRF8 expression kinetics during artificially induced exhaustion. **(b)** IRF8 geometric mean fluorescence intensity (gMFI) in P14^+^ CD8 T cells recovered from livers of experiment presented in Fig. 4 a with fold change compared with baseline indicated in brackets. *n* = 8 mice per condition, data from two pooled independent experiments. **(c)** ATAC-seq tracks at the *Irf8* locus of LCMV cl13 days 8 and 30 after infection P14^+^ T cells and TILs. Purple highlights indicate ATAC peaks decreasing in LCMV cl13 day 30 compared with LCMV cl13 day 8 P14^+^ T cells. TF-binding regions in CD8^+^ T cells (https://chip-atlas.org) are shown in green. **(d)** ATAC-seq and Cut&Tag (H3K27ac, GEO: GSE235007), tracks at the Tox locus of TILs and LCMV cl13 P14^+^ T cells. TF-binding regions in CD8^+^ T cells (https://chip-atlas.org) are shown in green. **(e and f)** IRF8 kinetics of enriched CD8 T cells activated with anti-CD3 and anti-CD28 antibodies with addition of IFNγ (e), IFNβ, or IL-12 (f) during 72 h compared with control (only IL-2). *N* = 3 biological replicates, e and f are two independent experiments. **(g)** IRF2, IRF4, and IRF7 gMFI of chronically TCR stimulated CD8 T cells (using protocol in a) with or without addition of IFNβ at different time points indicated in the figure. *N* = 6 biological replicates, representative data from three independent experiments. Two-way ANOVA comparing means within each condition was used to analyze data in b. One-way ANOVA was used in g. *P < 0.05, **P < 0.01, and ***P < 0.001.

We next investigated the regulation of IRF8 expression in CD8^+^ T cells. Previous studies have shown that IRF8 is induced by TCR engagement in CD8^+^ T cells (Miyagawa et al., 2012; Nelson et al., 1996; Sun et al., 2016). We reproduced these findings by activating CD8^+^ T cells with αCD3 and αCD28 antibodies *in vitro*, observing strong IRF8 upregulation during the first 72 h of activation (Fig. 3 a). To understand whether IRF8 expression was maintained during prolonged TCR engagement, we took advantage of an *in vitro* chronic TCR stimulation model in which CD8^+^ T cells undergo multiple rounds of restimulation with αCD3 antibodies (Fig. S2 a). Chronically stimulated cells gradually lost SLAMF6, upregulated multiple inhibitory receptors, such as PD-1, TIM3, and LAG3, and acquired granzyme B as well as TOX expression, suggesting the establishment of an exhausted-like phenotype (Fig. 3 b). Compared with resting CD8^+^ T cells (activated for 3 days and subsequently cultured with IL-7 and IL-15), chronically stimulated CD8^+^ T cells showed higher levels of IRF8, indicating that IRF8 expression was maintained during prolonged TCR stimulation (Fig. 3 c).

To test whether IRF8 expression was driven by TCR triggering *in vivo*, we co-transferred *in vitro* preactivated P14 and OT-I TCR-transgenic CD8^+^ T cells, recognizing the gp33 and the chicken OVA-derived OVA_257–264_ epitope, respectively, into B6 mice bearing B16-gp33 and B16-OVA tumors on opposite flanks (Fig. 3 d). At the time of adoptive transfer, both P14 and OT-I cells expressed similarly high levels of CD44, CD25, PD-1, and IRF8 (Fig. 3 e). 7 days after transfer, B16-gp33 tumor associated P14 TILs (tumor-specific) displayed an exhausted phenotype, with higher expression of PD-1 and TOX compared with the non-tumor–specific OT-I TILs (Fig. 3 f). Conversely, B16-OVA tumor-associated OT-I TILs (tumor-specific) showed higher PD-1 and TOX expression compared with P14 TILs (Fig. 3 g). Despite similar IRF8 expression levels in P14 and OT-I cells at the moment of co-transfer, IRF8 OE was exclusively observed in tumor-specific CD8^+^ T cells, while bystander cells expressed low IRF8 levels (Fig. 3, f and g), similar to cells that had been rested *in vitro* (Fig. 3 c). Together, these results show that TCR stimulation is required to induce and maintain IRF8 expression in tumor-associated CD8^+^ T cells.

### IRF8 is epigenetically repressed in CD8^+^ T cells responding to chronic viral infection

**Figure 4.**
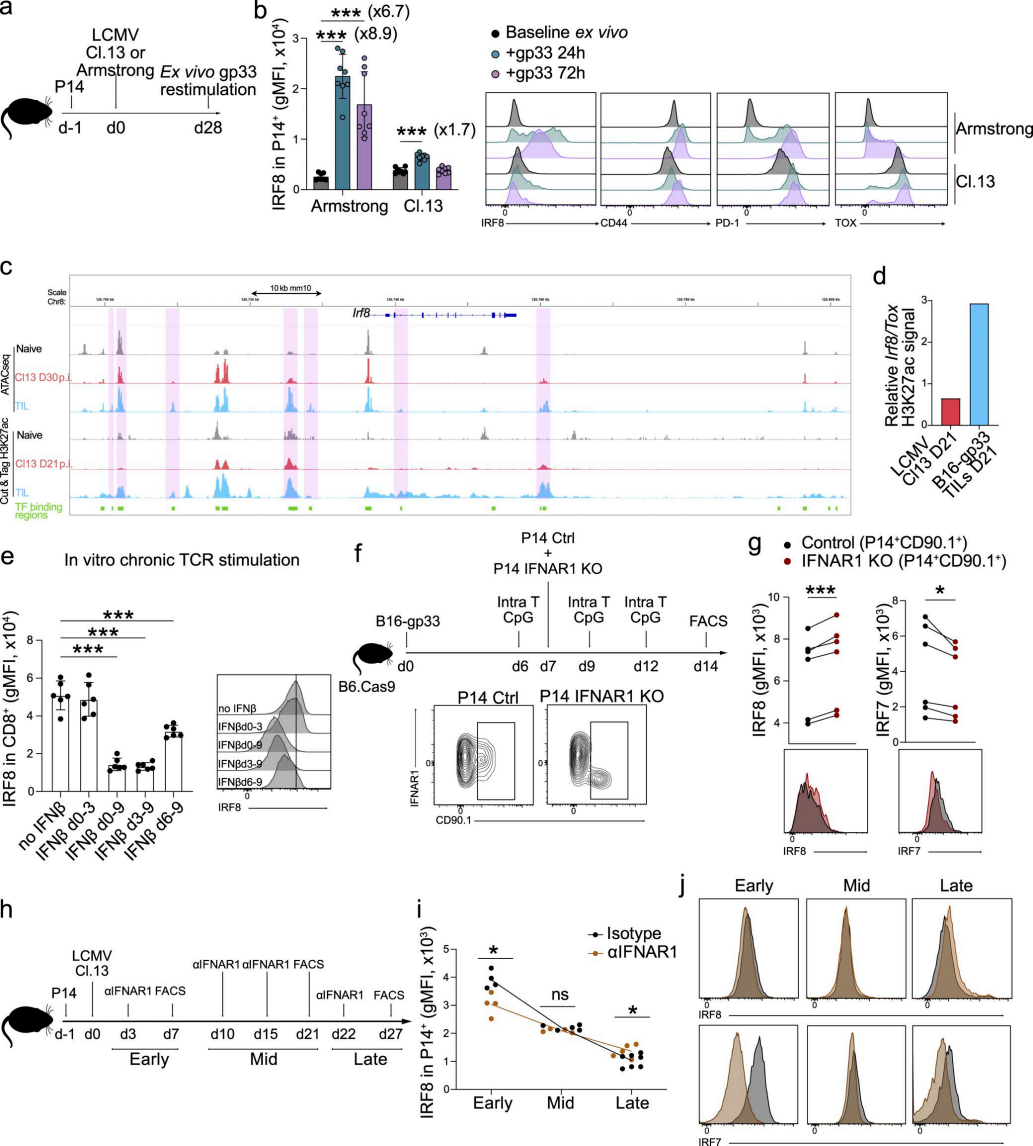
**IRF8 is epigenetically repressed in virus-specific CD8** ^**+**^ **T cells during chronic infection. (a)** Schematic representation for the experimental procedure used to assess the capacity of P14 CD8^+^ T cells recovered from acute LCMV Arm or chronic LCMV Cl13 infection to upregulate IRF8 after *ex vivo* gp33 stimulation. **(b)** IRF8 levels in P14^+^ CD8 T cells obtained as described in a at *ex vivo* baseline, 24, and 72 h after restimulation with gp33 peptide (left) with representative histograms showing IRF8, CD44, PD-1, and TOX expression (right). *n* = 8 mice per condition, data pooled from two independent experiments. Fold-change respective to baseline is indicated in brackets. **(c)** ATAC-seq and Cut&Tag (H3K27ac, GEO: GSE235007) tracks and TF-binding regions (mouse CD8^+^ T cells; https://chip-atlas.org) at the *Irf8* locus. Purple highlights indicate ATAC peaks increased in TILs compared with LCMV cl13 P14^+^ T cells. **(d)** Signal quantification for H3K27ac tracks represented as relative *Irf8*/*Tox* ratio. **(e)** IRF8 levels of *in vitro* chronically TCR stimulated CD8 T cells cultured with or without IFNβ during the indicated period with representative histogram. *n* = 6 biological replicates, representative data from three independent experiments. **(f)** Schematic representation of experimental procedure used to assess the capacity of type I IFN to downregulate IRF8 levels in CD8^+^ TILs. P14-Cas9 IFNAR1 KO and control-transduced P14-Cas9 cells were co-transferred in B16-gp33–bearing B6-Cas9 mice that received intratumoral CpG injection at the indicated time points as described in the Materials and methods section. At the bottom, FACS plots showing expression of IFNAR1 and CD90.1, expressed only in transduced cells, in P14 control and P14 IFNAR1 KO cells. **(g)** Flow cytometric analysis of IRF8 and IRF7 levels in P14^+^CD90.1^+^ IFNAR1 KO or control transduced CD8 TILs with representative FACS histograms. *n* = 6, data pooled from two independent experiments. **(h)** Schematic representation showing experimental procedure used to assess IRF8 levels during chronic LCMV Cl13 infection upon αIFNAR1 antibody treatment at the indicated time point, as described in the Materials and methods. **(i and j)** IRF8 quantification (i) at the indicated time point as illustrated in h with representative FACS histograms for IRF8 and IRF7 expression (j). *n* = 4–5 mice per condition at each time point. Representative data from three independent experiments are shown. In b statistical analysis was done with two-way ANOVA with comparing means within each group. In e statistical analysis was done with one-way ANOVA comparing means with control mean (no IFNβ). In g, statistical analysis was done by paired two-tailed Student’s *t* test. In h, statistical analysis was done by unpaired two-tailed Student’s *t* test. *P < 0.05 and ***P < 0.001.

Since persistent TCR engagement is a shared core feature of cancer and chronic infection, we speculated that IRF8 was repressed during chronic infection. To test this hypothesis, we transferred naive P14 cells into Vβ5 mice that were then infected with LCMV cl13 or into B6 mice infected with LCMV Armstrong (Arm), which results in acute resolved infection, and collected cells 28 days later (Fig. 4 a). We stimulated the recovered P14 cells for 24 or 72 h with gp33 peptide to assess their capacity to upregulate IRF8 expression *ex vivo*. P14 cells from the acute resolved LCMV Arm infection highly upregulated IRF8 at 24 h (8.9-fold) and 72 h (6.7-fold) after restimulation. In comparison, P14 cells from chronically infected mice displayed considerably lower upregulation (1.7-fold), despite increased expression of CD44, PD-1, and TOX (Fig. 4 b and Fig. S2 b).

We next determined the accessibility of the *Irf8* locus based on our scATAC-seq (single-cell assay for transposase-accessible chromatin with sequencing) from tumor-associated P14 TILs (Tille et al., 2023) with published scATAC-seq data from chronic infection–derived P14 cells (Giles et al., 2022). While the accessibility of the *Tox* locus was comparable (Fig. S2 d), several regions close to the *Irf8* locus, which overlap with TF–binding regions determined thanks to mouse CHIPseq CD8^+^ T cell datasets from CHIP-Atlas (see Materials and methods), displayed decreased accessibility in chronic infection-derived P14 cells (Fig. 4 c). The reduced accessibility correlated with reduced H3K27ac deposition, explaining the substantially reduced *Irf8* transcription in chronic infection–derived P14 cells (Fig. 4, c and d) (Ma et al., 2025). Interestingly, the scATAC-seq profile of P14 cells displayed lower accessibility of the *Irf8* locus during the chronic phase of infection compared with acute phase of the infection (day 8). These data suggested that IRF8 expression underwent epigenetic repression during chronic infection (Fig. S2 c).

**Figure S3.**
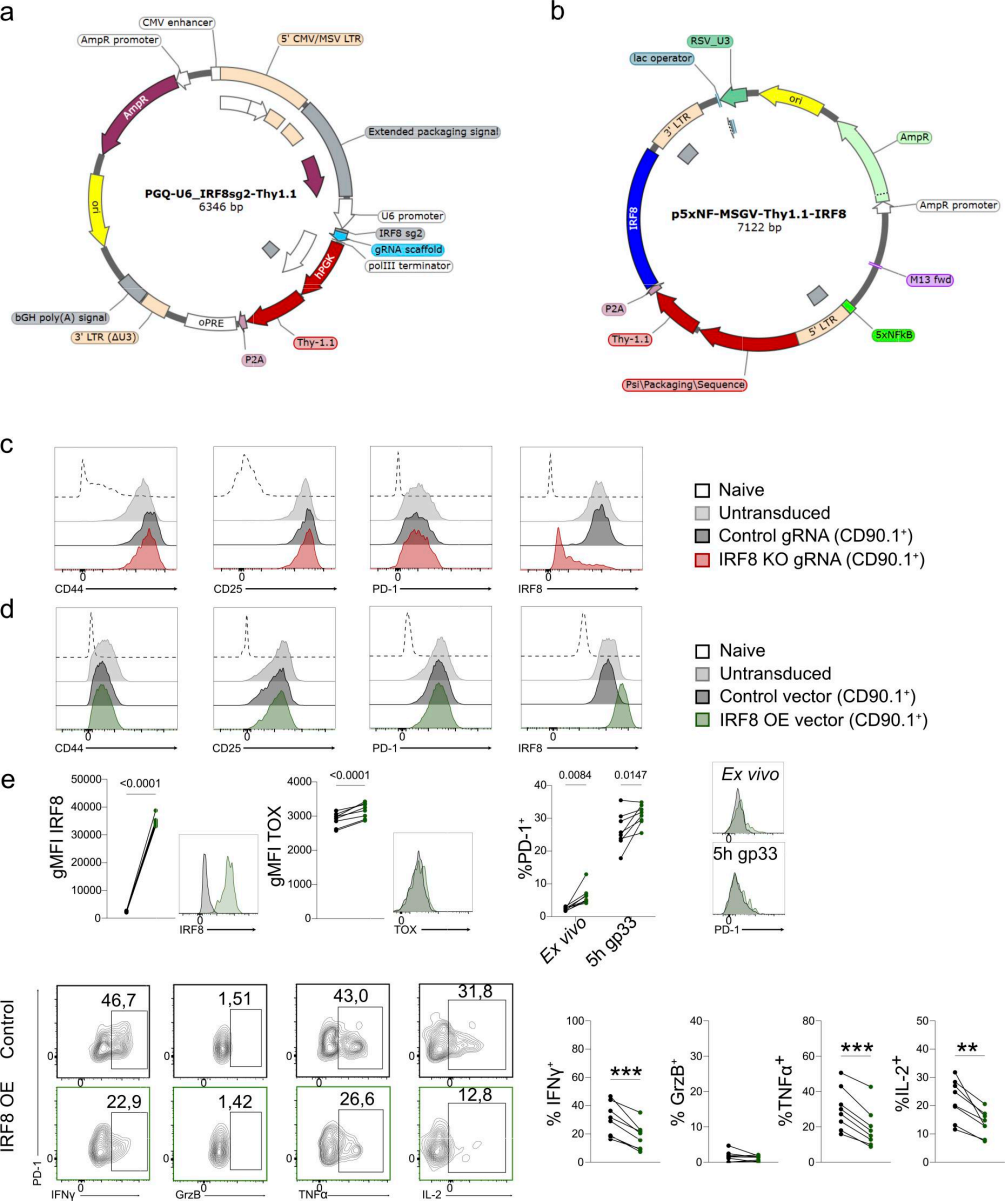
**IRF8 level modulation did not change activation quality in CD8** ^**+**^ **T cells. (a)** Representation of the retroviral vector PQG-Thy1.1 used to induce KO in P14^+^CD8 T cells expressing the Cas9 endonuclease (see Materials and methods). The specific sgRNA sequence targeting the gene of interest (in this case IRF8) was inserted in front of the gRNA scaffold sequence under the control of a U6 promoter and followed by Thy1.1 (CD90.1) sequence, used as transduction marker, under the control of hPGK promoter. **(b)** Representation of the retroviral vector pMSGV-Thy1.1 used to induce IRF8 OE. The full IRF8 coding-sequence was inserted after the Thy1.1 sequence, separated by a P2A cassette. The control used was the same vector without the *Irf8* sequence (empty). **(c and d)** Representative FACS histograms showing CD44, CD25, PD-1, and IRF8 expression in naïve, untransduced, control transduced, P14-Cas9 IRF8 KO transduced (c), or P14 IRF8 OE transduced (d) CD8^+^ T cells 48 h after activation with retroviral transduction being done at 24 h after activation, as described in the Materials and methods. **(e)** These data complement experiments presented in Fig. 5 e, IRF8 and TOX gMFI, percentage of PD-1^+^ before and after *ex vivo* peptide restimulation, and percentage of IFNγ^+^, GrzB^+^, TNFα^+^, and IL-2^+^ with representative FACS plots in IRF8 OE compared with control P14 cells recovered from LNs from experiment illustrated in Fig. 6, k–p. Representative data from two independent experiments (*n* = 8). Statistical analysis was done with paired two-tailed Student’s *t* test. **P < 0.01 and ***P < 0.001.

Several factors differ between chronic infection and cancer and may regulate IRF8 accessibility. Among them, increased inflammation with increased IFN signaling has been described during chronic infection (Teijaro et al., 2013; Wilson et al., 2013). We tested the effect of IFNγ, described to induce IRF8 concomitant to TCR engagement (Nelson et al., 1996), and of IFNβ as hallmark for chronic type I IFN exposure during chronic infections on IRF8 expression during the first 72 h after CD8 T cell activation. While IFNγ and IL-12 did not alter IRF8 levels, IFNβ strongly repressed IRF8 induction (Fig. S2, e and f), suggesting a negative regulation of IRF8 expression by type I IFN. To confirm the effect of IFNβ during chronic TCR stimulation, we used our *in vitro* chronic TCR stimulation assay and added IFNβ at different time points. We observed increased levels of IRF7, an IFN-responsive TF (Fig. S2 g). In contrast, IRF8 protein levels did not change when IFNβ was present form from day 0 to 3 but were drastically decreased when IFNβ was present after day 3 (e.g., day 0 to day 9, day 3 to day 9, or day 6 to day 9), showing that type I IFN repressed IRF8 during chronic TCR stimulation (Fig. 4 e). To assess the effect of type I IFN *in vivo*, we transduced P14 cells from CD4-Cre STOP^fl/fl^-Cas9 mice (hereafter P14-Cas9) with a retroviral vector containing a sgRNA-targeting *Ifnar1* (IFNAR1 KO) or a nontargeting sgRNA (control) (Fig. S3 a). IFNAR1 KO and control P14 cells were then co-transferred in a 1:1 ratio into B16-gp33-tumor bearing Rosa26-Cas9 knock-in mice (hereafter B6-Cas9) (Fig. 4 f). The use of B6-Cas9 mice as host prevents the rejection of P14-Cas9 cells due to the tolerance of these mice to the Cas9 protein. Mice were intratumorally injected with CpG to induce the production of type I IFN (Wang et al., 2016). IFNAR1 KO P14 cells showed significantly higher IRF8 levels compared with control P14 cells, while IRF7 levels were decreased in IFNAR1 KO P14 cells, confirming the CD8^+^ T cell–intrinsic suppression of IRF8 by type I IFN signaling (Fig. 2 g).

To investigate whether type I IFN impacts IRF8 expression during chronic infection, we injected αIFNAR1-blocking antibodies at different time points during the course of infection. While late blockade (from day 22 to 27) significantly increased IRF8 expression compared with isotype control antibody-treated mice, blockade at mid time point (from day 10 to 20) showed no significant difference, while early blockade decreased IRF8 expression (Fig. 4, i and j). The gradual decrease in IRF8 expression in control mice over time is consistent with our observation that the *Irf8* locus is less accessible on day 30 compared with day 8 after infection, suggesting progressive IRF8 repression over the course of infection (Fig. 4, c, i, and j; and Fig. S2 c).

Taken together, these data show that IRF8 expression in CD8^+^ T cells is driven and maintained by TCR engagement. While it is maintained in the TME, it is repressed during chronic infection through epigenetic remodeling of the *Irf8* locus. Our results suggest that type I IFN participates at least partially in the downregulation of IRF8 at later stages during chronic infection. However, additional mechanisms likely contribute to IRF8 repression in CD8^+^ T cells.

### IRF8 expression in CD8^+^ T cells promotes exhaustion and reduces anti-tumor function

**Figure 5.**
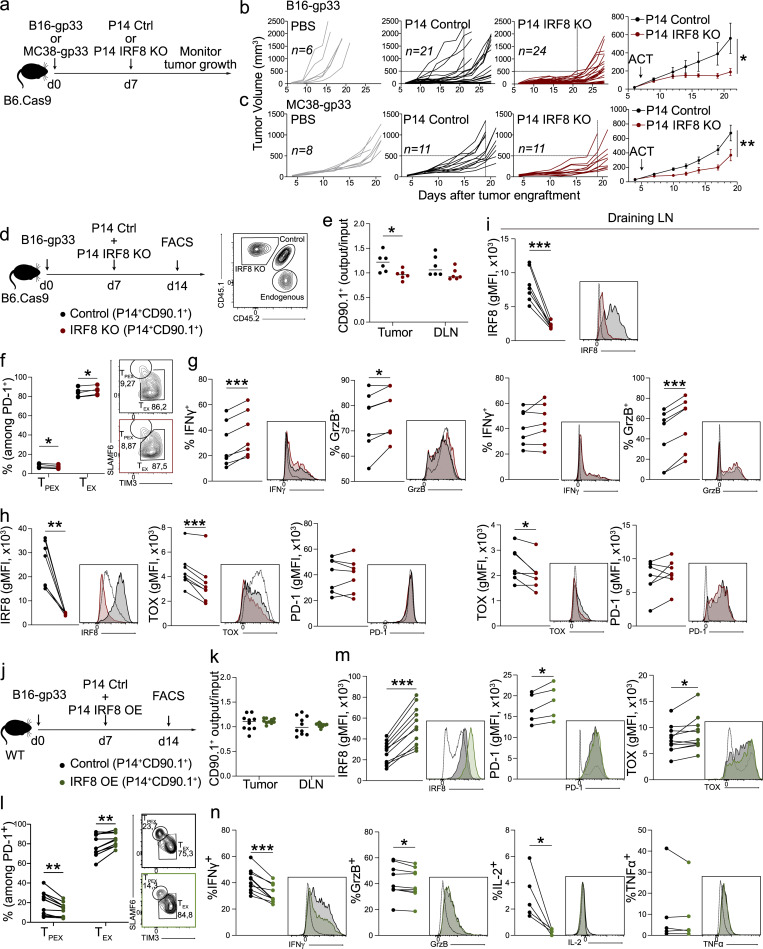
**IRF8 expression in CD8** ^**+**^ **T cells promotes exhaustion and reduces anti-tumor function. (a)** Schematic representation of experimental procedure used to assess tumor control capacity of IRF8 KO CD8^+^ T cells. **(b and c)** Tumor growth curves in B6-Cas9 mice that received a mock PBS injection, control-transduced P14 CD8 T cells, or IRF8 KO P14 CD8 T cells 7 days after B16-gp33 (b) or MC38-gp33 (c) tumor engraftment and mean of control and IRF8 KO P14 CD8 T cells transferred mice (bars show SEM). Numbers of mice for each condition are indicated in the figure, and data are issued from two pooled independent experiments in b and representative of two independent experiments in c. **(d)** Schematic representation showing experimental procedure used to address IRF8 KO effect in P14-Cas9 CD8^+^ TILs, with FACS plots showing gating strategy for differentiating KO, control, and endogenous cells among CD8^+^ cells in the B16-gp33 tumor of B6-Cas9 mice. **(e)** Recovered (output) versus injected (input) CD90.1^+^ control or KO CD8^+^ T cells represented as ratio output/input in tumor and DLN from co-transfer experiment illustrated in d. **(f)** Percentages of T_PEX_ and T_EX_ populations in IRF8 KO and control-transduced cells. **(g–i)** Flow cytometric analysis of IRF8, TOX, and PD-1 (gMFI) and IFNγ^+^ and GrzB^+^ (percentage) in P14 IRF8 KO and P14 control CD8^+^ T cells in tumor and DLN. Representative data from three independent experiments (*n* = 7). **(j)** Schematic representation showing experimental procedure used to address IRF8 OE effect in P14 CD8^+^ TILs in B16-gp33 tumors in WT B6 mice. **(k)** Recovered (output) versus injected (input) CD90.1^+^ control or OE CD8^+^ T cells represented as ratio output/input in tumor and DLN from co-transfer experiment illustrated in j. **(l)** Percentages of T_PEX_ and T_EX_ populations in IRF8 OE and control-transduced cells. **(m and n)** Flow cytometric analysis of IRF8, TOX, and PD-1 (gMFI), and IFNγ^+^, GrzB^+^, IL-2^+^, and TNFα^+^ (percentage) in P14 IRF8 OE and P14 control CD8^+^ T cells in tumor. Data from two pooled independent experiments (*n* = 10). Statistical analysis for b and c was done by repeated measures two-way ANOVA. In f–i and l–n, statistical analysis was done with paired two-tailed Student’s *t* test. Statistical analysis in e and k was done with two-tailed Student’s *t* test. *P < 0.05, **P < 0.01, and ***P < 0.001.

Since IRF8 expression strongly correlated with exhaustion of tumor-associated CD8^+^ T cells where we inferred specific transcriptional activity, we tested whether IRF8 contributed to CD8^+^ T cell exhaustion. We performed adoptive cell transfers (ACTs) of P14-Cas9 control or P14-Cas9 IRF8 KO cells into B6-Cas9 mice 7 days after B16-gp33 or MC38-gp33 tumor inoculation (Fig. 5 a). Mice injected with P14 IRF8 KO cells displayed a significant delay in tumor growth compared with mice that received P14 control cells in both tumor models tested (Fig. 5, b and c). To investigate the effect of IRF8 ablation in CD8^+^ TILs, we co-transferred P14 IRF8 KO and P14 control cells in a ratio 1:1 into B16-gp33–bearing B6-Cas9 mice (Fig. 5 d). Based on differential expression (DE) of CD45.1 and CD45.2, we could discriminate between endogenous CD8^+^ T cells (CD45.2^+^) and transferred P14-Cas9 cells transduced with IRF8-targeting or control sgRNA (CD45.1^+^ and CD45.1^+^CD45.2^+^). This strategy allowed a direct and unbiased comparison of the cell-intrinsic effects of IRF8 ablation in CD8^+^ T cells exposed to the same TME, controlling as well for tumor size effect. At the time of the adoptive transfer, IRF8 KO and control P14 cells displayed a similar activation profile based on the expression of CD44, CD25, and PD-1 (Fig. S3 c). Transduced cells were followed thanks to the expression of CD90.1. 7 days after co-transfer, IRF8 KO cells accumulated slightly less efficiently in the tumor compared with control cells, but not in the draining LN (DLN) (Fig. 5 e). When analyzing T_PEX_ (SLAMF6^+^ TIM-3^−^) versus T_EX_ (SLAMF6^−^ TIM-3^+^), we observed a weak but significant increase in the proportion IRF8 KO T_EX_ (Fig. 5 f). Nevertheless, IRF8 KO cells displayed significantly lower levels of TOX with no effect on PD-1 expression (Fig. 5 h), together with an increase in IFNγ and GrzB production upon *ex vivo* peptide restimulation compared with control cells from the same tumor (Fig. 5 g). We observed similar effects in the DLN, suggesting that IRF8 ablation alleviates exhaustion features in tumor-associated CD8^+^ T cells (Fig. 5 i).

To further investigate the capacity of IRF8 to regulate T cell exhaustion, we overexpressed IRF8 by transducing P14 cells with a retroviral vector encoding *Irf8* (hereafter P14 IRF8 OE) or an empty control vector (Fig. S3 b). We co-transferred the cells in a 1:1 ratio into B6 mice 7 days after B16-gp33 engraftment and analyzed the tumor 7 days later (Fig. 5 j). IRF8 OE and control cells did not differ in terms of activation at the time of transfer (Fig. S3 d). OE of IRF8 did not change the accumulation of P14 cells in the tumor and the DLN (Fig. 5 k) but significantly impacted the proportions of T_PEX_ and T_EX_ with an increase in the T_EX_ population, suggesting that IRF8 promotes terminal exhaustion (Fig. 5 l). We observed a significant increase in TOX and PD-1 expression together with a decrease in IL-2, GrzB, and IFNγ production after restimulation of P14 IRF8 OE compared with P14 control cells, mirroring the results found with IRF8 KO CD8^+^ T cells (Fig. 5, d–i). Collectively, these results showed that IRF8 promoted exhaustion and reduced the effector functions of tumor-associated CD8^+^ cells. Indeed, ACT using IRF8 KO CD8^+^ T cells ameliorated tumor control.

### IRF8 has limited effect on exhausted CD8^+^ T cells during chronic infection but limits effector function in absence of persistent TCR stimulation

**Figure 6.**
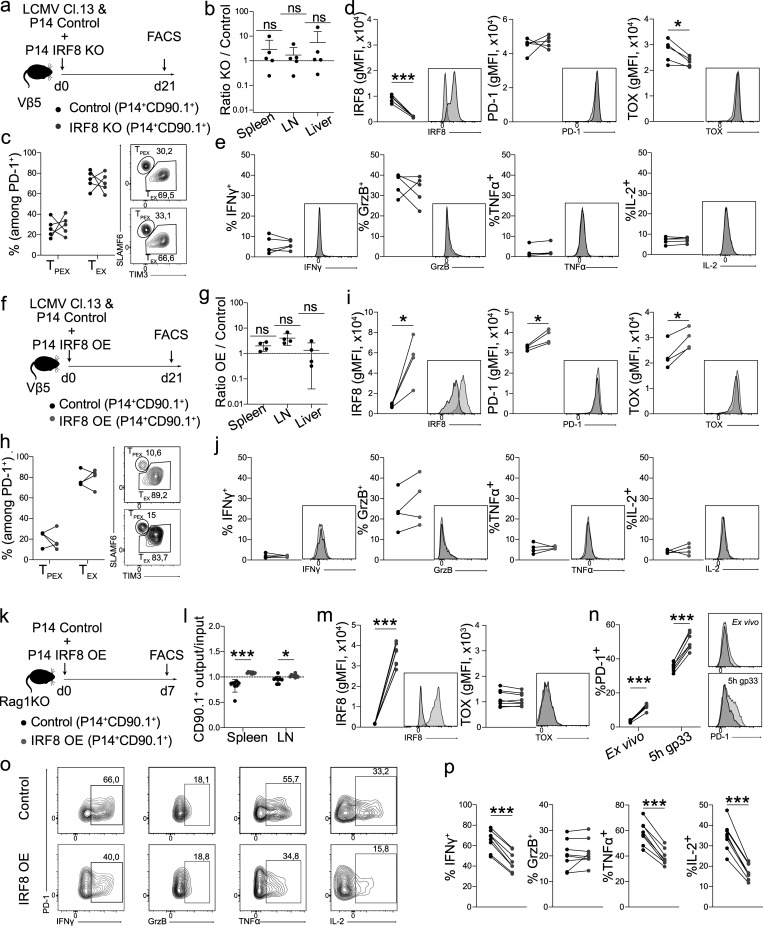
**IRF8 has limited effect on exhausted CD8** ^**+**^ **T cells during chronic infection but limits effector function in absence of persistent TCR stimulation. (a)** Schematic representation showing experimental procedure used to address IRF8 KO effect in P14-Cas9 CD8^+^ T cells during chronic LCMV Cl13 infection in Vβ5 mice. **(b)** Accumulation of IRF8 KO cells compared with control cells represented as ratio KO/control in spleen, LN, and liver at day 21 after infection. **(c–e)** Percentage of T_PEX_ and T_EX_ (c), percentage of IFNγ^+^, GrzB^+^, TNFα^+^, and IL-2^+^ (e), and gMFI of IRF8, PD-1, and TOX (d) in IRF8 KO compared with control P14 cells from experiments illustrated in a. Representative experiment from two independent experiments (*n* = 5). **(f)** Schematic representation showing experimental procedure used to address IRF8 OE effect in P14 CD8^+^ T cells during chronic LCMV Cl13 infection in Vβ5 mice. **(g)** Accumulation of IRF8 KO cells compared with control cell represented as ration KO/control in spleen, LN, and liver at day 21 after infection. **(h–j)** Percentage of T_PEX_ and T_EX_ (h), percentage of IFNγ^+^, GrzB^+^, TNFα^+^, and IL-2^+^ (j), and gMFI of IRF8, PD-1, and TOX (i) in IRF8 KO compared with control P14 cells from experiment illustrated in f. Representative experiment from two independent experiments (*n* = 4). **(k)** Schematic representation showing experimental procedure used to address IRF8 OE effect in P14 CD8^+^ T cells in Rag1KO mice. **(l)** Recovered (output) versus injected (input) CD90.1^+^ control or OE CD8^+^ T cells represented as ratio output/input in LN and spleen from co-transfer experiment illustrated in k. **(m–p)** IRF8 and TOX gMFI (m), percentage of PD-1^+^ before and after *ex vivo* peptide restimulation (n), and percentage of IFNγ^+^, GrzB^+^, TNFα^+^, and IL-2^+^ (p) with representative FACS plots (o) in IRF8 OE compared with control P14 cells from experiment illustrated in k. Data from two pooled independent experiments (*n* = 8). In b and g, statistical analysis was done with a *t* test comparing the means with a theoretical value of 1, representing a fold change of 1. In c–e and m–p, statistical analysis was done with paired two-tailed Student’s *t* test. Statistical analysis in l was done with two-tailed Student’s *t* test. *P < 0.05 and ***P < 0.001.

Our results indicated that IRF8 is specifically upregulated in the tumor, while CD8^+^ T cells responding to chronic viral infection expressed low levels of IRF8. To examine the role of IRF8 in chronic viral infection, we co-transferred P14 IRF8 KO together with control transduced cells into Vβ5 mice and infected them with LCMV cl13 (Fig. 6 a). At day 21 after infection, similar numbers of P14 IRF8 KO and P14 control cells were found in the spleen, LN, and liver, without significant changes in the proportions of T_PEX_ and T_EX_ cells (Fig. 6, b and c). IRF8 levels in IRF8 KO P14 cells were lower than in control P14 cells, showing that CD8^+^ T cells responding to chronic infection express low IRF8 levels. Consistent with this, P14 IRF8 KO cells showed a modest but significant decrease in TOX expression with no effect on PD-1 expression (Fig. 6 d). However, we did not observe changes in cytokine production upon *ex vivo* restimulation (Fig. 6 e). We also overexpressed IRF8 in P14 cells before transfer into LCMV cl13-infected mice (Fig. 6 f). P14 IRF8 OE cells showed a trend to increased accumulation in secondary LNs and the spleen but not the liver of LCMV cl13-infected mice. There were no changes in the proportions of T_PEX_ and T_EX_ cells (Fig. 6, g and h). Increased IRF8 expression resulted in a slight but significant increase in PD-1 and TOX expression, but did not further reduce the already low capacity to produce cytokine upon restimulation (Fig. 6, i and j). Alternatively, IRF8 might not be able to integrate into the regulatory network as other TFs might dominate the regulatory landscape.

In view of these findings, we next wondered whether IRF8 OE impacted the phenotype of CD8^+^ T cells when these cells expand in the absence of persistent antigen stimulation, but rather driven by homeostatic cytokines. To address this, we co-transferred P14 IRF8 OE and P14 control into *Rag1*^*−/−*^ mice and analyzed splenocytes 7 days later (Fig. 6 k). We recovered significantly higher numbers of IRF8 OE compared with control cells in both the spleen and LNs of *Rag1*^*−/−*^ mice (Fig. 6 l). Interestingly, P14 IRF8 OE cells showed increased PD-1 levels compared with control cells, while TOX level did not change in the spleen but were slightly increased in LN (Fig. 6 m and Fig. S3 e). Upon *ex vivo* restimulation with gp33 peptide, IRF8 OE cells upregulated PD-1 more strongly compared with the control cells (Fig. 6 n and Fig. S3 e). Moreover, IRF8 OE cells displayed strongly impaired effector functions with lower capacity to produce IL-2, TNFα, and IFNγ (Fig. 6, o and p; and Fig. S3 e), showing that OE of IRF8 is sufficient induce some features associated with exhaustion in CD8^+^ T cells even in absence of persistent TCR stimulation, similarly to what has been described for TOX (Khan et al., 2019). Altogether, these results show that IRF8 promotes TOX expression and hinders effector functions.

### Deletion of IRF2, IRF4, and IRF8 attenuates exhaustion in tumor specific CD8^+^ T cells

**Figure 7.**
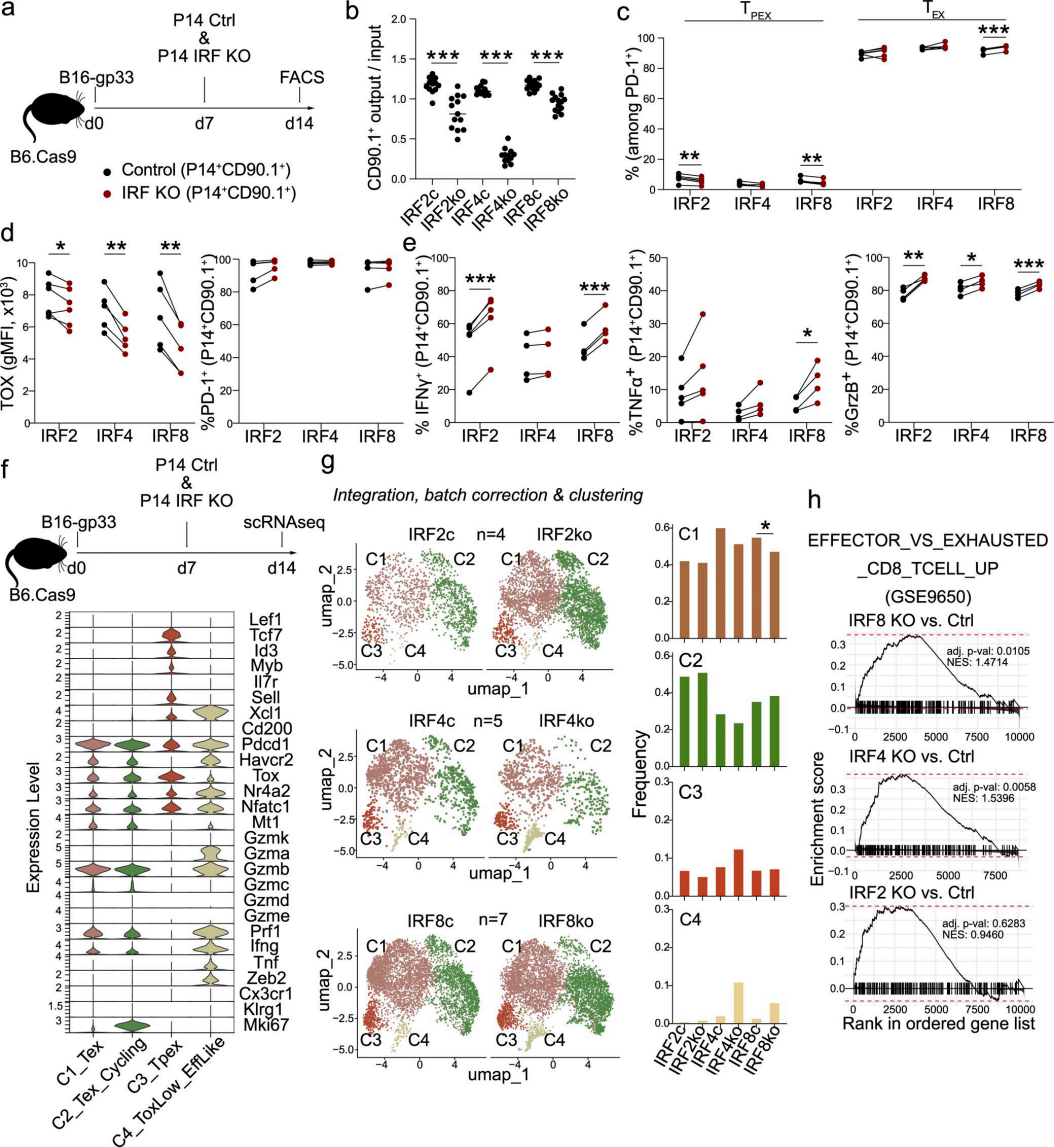
**Deletion of IRF2, IRF4, and IRF8 attenuates exhaustion in tumor-specific CD8** ^**+**^ **T cells. (a)** Schematic representation of experimental procedure used to assess effects of IRF2, IRF4, and IRF8 KO in P14 CD8^+^ TILs from B16-gp33–bearing B6-Cas9 mice. **(b)** Recovered (output) versus injected (input) CD90.1^+^ control (c) or KO (ko) CD8^+^ T cells from co-transfer experiments of IRF2, 4, or 8 KO with control-transduced P14^+^ CD8 T cells represented as ratio output/input 7 days after co-transfer. Data were pooled from three independent experiments (*n* = 13 for IRF2 KO, *n* = 12 for IRF4 KO, and *n* = 14 for IRF8 KO). **(c)** Percentages of T_PEX_ and T_EX_ populations in IRF KO and control-transduced cells. **(d and e)** TOX gMFI and PD-1^+^ percentage (d) and percentage of IFNγ^+^, TNFα^+^, and GrzB^+^ (e) after restimulation with gp33 peptide and of P14^+^CD90.1^+^ control and IRF KO CD8 T cells from co-transfer experiment illustrated in a. Representative data from three independent experiments (*n* = 6 for IRF2 KO and *n* = 5 for IRF4 and IRF8 KO). **(f)** Schematic representation of experimental procedure used to assess transcriptional changes of IRF2, IRF4, and IRF8 KO in P14 CD8^+^ TILs from B16-gp33–bearing B6-Cas9 mice (top) and violin plots displaying expression levels of markers spanning progenitor, exhaustion, effector, and proliferative programs in the four clusters defined in g. **(g)** UMAP plots defining clustering after integration and batch correction for each P14 IRF KO and their respective co-transferred control CD8 TILs derived from scRNA-seq data and clustered using Seurat, with quantification for each of the four clusters in control versus IRF KO. *n* = 4 for IRF2 KO, *n* = 5 for IRF4 KO, and *n* = 7 for IRF8 KO. **(h)** GSEA of pseudobulk from scRNA-seq for the indicated IRF KO versus control using gene set effector versus exhausted CD8 T cells UP. GEO accession: GSE9650. Adjusted P value (adj. P val) and normalized enrichment score (NES) are shown for each comparison. In b, statistical analysis was done by two-tailed Student’s *t* test. In c–e and g, statistical analysis was done with paired two-tailed Student’s *t* test. *P < 0.05, **P < 0.01, and ***P < 0.001.

**Figure S4.**
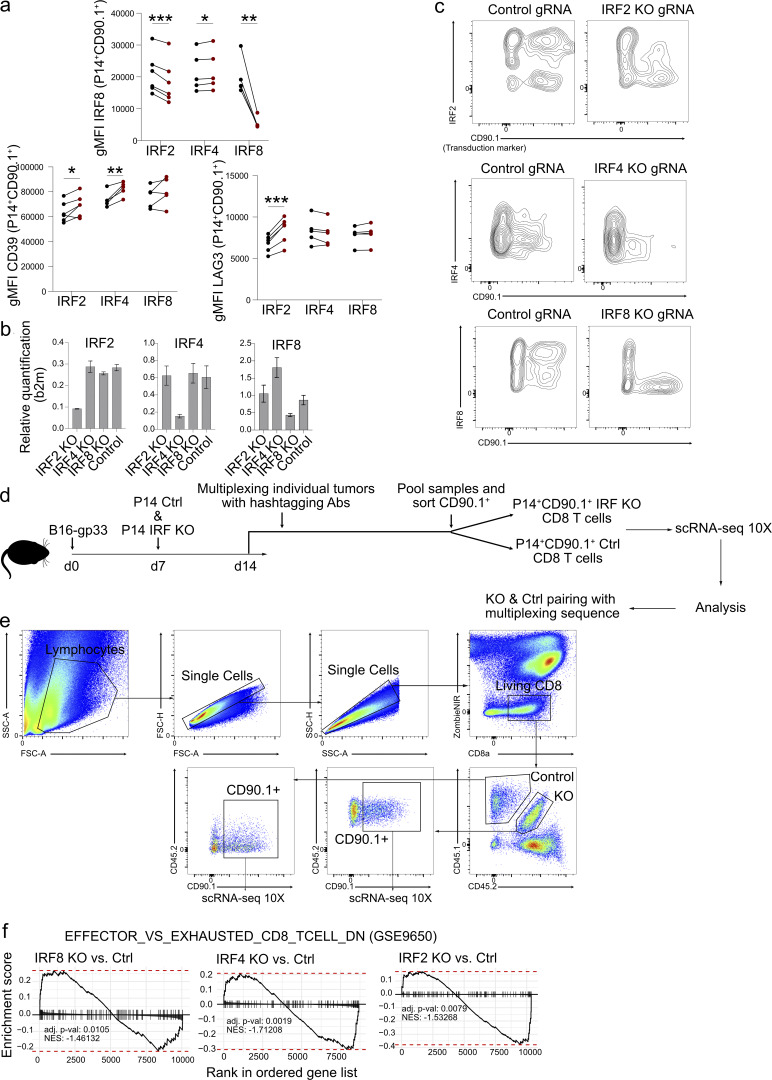
**Comparison of IRF2, IRF4, and IRF8 KO in CD8 TILs. (a)** Expression of IRF8, LAG3, and CD39 in IRF KO P14^+^CD90.1^+^ CD8 TILs compared with their co-transferred control-transduced counterpart. **(b)** IRF2, IRF4, and IRF8 KO validation by qRT-PCR performed in the different transduced CD90.1^+^ CD8 T cells after transduction and FACS sorting. **(c)** IRF2, IRF4, and IRF8 KO validation by FACS performed in the different transduced CD90.1^+^ CD8 T cells recovered from tumors. **(d)** Schematic representation of experimental strategy used for scRNA-seq on *ex vivo* co-transferred IRF KO P14^+^CD90.1^+^ CD8 TILs with multiplexed hashtagging before FACS sorting and sequencing for paired sample analysis. **(e)** Representative gating strategy used for FACS-sorting hashtagged IRF KO and control-transduced transferred P14 CD8 T cells from B16-gp33 tumors for scRNA-seq. **(f)** GSEA of pseudobulk from scRNA-seq for the indicated IRF KO versus control using gene set effector versus exhausted CD8 T cells down. GEO accession: GSE9650. Adjusted P value (adj. P val) and normalized enrichment score (NES) are shown for each comparison. *P < 0.05, **P < 0.01, and ***P < 0.001.

Recently, IRF2 in tumors (Lukhele et al., 2022) and IRF4 during chronic infection and in tumors (Man et al., 2017; Seo et al., 2021; Yan et al., 2023) have been shown to regulate CD8^+^ T cell exhaustion. We hypothesized that these IRF family members may play a convergent/redundant role in the regulation of exhaustion. To address this point, we directly compared KO of IRF2 and IRF4 with IRF8 KO in CD8^+^ TILs. We performed co-transfers of IRF2, IRF4, or IRF8 KO P14 cells with control transduced P14 cells into B16-gp33–bearing mice (Fig. 7 a and Fig. S4, b and c). The recovered transduced (CD90.1^+^) population was constant in all conditions in the case of the control transduction. In contrast, compared with the injected T cell product, the recovery of transduced (CD90.1^+^) population was decreased for the different KO, with minimal changes in the proportions of T_PEX_ versus T_EX_ populations (Fig. 7, b and c). IRF4 KO CD8^+^ T cells were drastically decreased in number, consistent with a critical role of IRF4 in the clonal expansion during infection (Man et al., 2017; Yao et al., 2013). IRF2 was also decreased, but to a lesser extent, similarly to IRF8 KO. TOX levels were reduced in IRF4 and IRF8 KO, and to a lesser extent in IRF2 KO (Fig. 7 d). Conversely, LAG3 or CD39 showed the opposite trend (Fig. S4 a) while PD-1 level remained elevated (Fig. 7 d). The functionality of IRF KO cells was partially improved, with an increase of IFNγ production in IRF2 and IRF8 KO cells, while GrzB and TNFα slightly increased in all IRF KOs upon restimulation (Fig. 7 e). Altogether, several IRF family members decrease TOX and increase effector functions in tumor-specific CD8^+^ T cells, with the strongest phenotype observed in IRF8 KO cells. These data suggest possible redundancy of IRF family members in anti-tumor–specific CD8^+^ T cell responses.

### Multiplexed scRNA-seq and CUT&RUN reveal convergence of IRF2, IRF4, and IRF8 on TOX-driven exhaustion in CD8^+^ TILs

To further investigate the role of the three TFs, we performed scRNA-seq on *ex vivo* TILs using the same experimental setting. P14 IRF KO CD8^+^ T cells were co-transferred with P14 control cells into B16-gp33–bearing mice. 7 days after co-transfer, tumors were processed, individually labeled with a hashtagging antibody, and pooled before FACS sorting. This protocol allowed us to directly compare the transcriptome of P14 IRF KO cells with P14 control cells derived from the same tumor (Fig. 7 f and Fig. S4, d and e). After batch integration and correction, we defined four populations of P14 cells based on unsupervised clustering together with the expression of marker genes spanning progenitor, exhaustion, effector, and proliferative programs. IRF KOs did not result in major changes in the cell distribution to the four clusters (Fig. 7, f and g). However, IRF4 and IRF8 KO resulted in the emergence of a TOX-low population with effector-like features (C4), while the terminally differentiated T_EX_ population (C1) declined (Fig. 7 g). Gene set enrichment analysis (GSEA) confirmed the increased overlap with genes associated with effector CD8^+^ T cells and reduced overlap with genes associated with exhausted CD8^+^ T cells (Fig. 7 h and Fig. S4 f).

**Figure 8.**
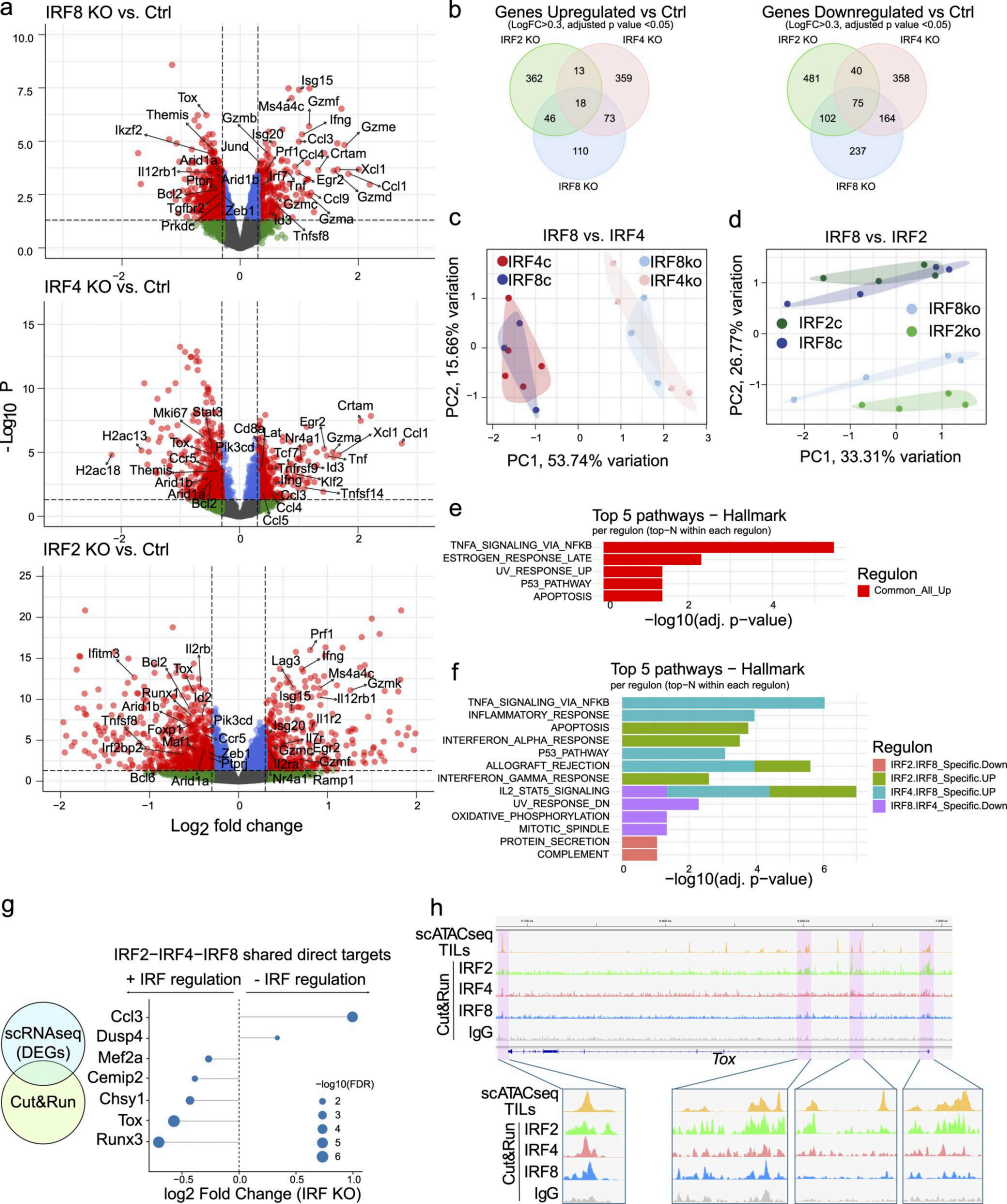
**Multiplexed scRNA-seq reveals convergence of IRF2, IRF4, and IRF8 on TOX-driven exhaustion in CD8** ^**+**^ **TILs. (a)** Volcano plots from scRNA-seq showing significant DEGs (LogFC>0.3, adjusted P value <0.05) in IRF8 KO (top), IRF4 KO (middle), and IRF2 KO (bottom) P14^+^ CD8 T cells compared with their co-transferred control-transduced counterpart (paired analysis using multiplexed hashtagging antibodies) from experiment in Fig. 7 f. **(b)** Venn diagrams showing the numbers of DEGs that are upregulated (left) and downregulated (right) in each IRF KO compared with control (threshold LogFC>0.3, adjusted P value <0.05). **(c and d)** PCA of scRNA-seq from IRF8 KO versus IRF4 KO (c) and IRF8 KO versus IRF2 KO (d) with their respective co-transferred control-transduced CD8 TILs issued from co-transfer experiments in Fig. 7 f. **(e)** Overrepresentation of pathways from MSigDB Hallmark gene sets across up- and downregulated genes common to IRF2, IRF4, and IRF8 KO. **(f)** Overrepresentation of pathways from MSigDB Hallmark gene sets across up- and downregulated genes common to IRF2 and IRF8 KO and to IRF4 and IRF8 KO. **(g)** Lollipop plot showing shared direct targets of IRF2, IRF4, and IRF8 based on integration of scRNA-seq data from above and CUT&RUN-seq data generated on *in vitro* chronically TCR-stimulated CD8^+^ T cells as described in the Materials and methods. Each point represents a gene, with the x-axis indicating log2 fold change (IRF8 KO versus control) and point size representing statistical significance (–log10 FDR). Genes are ordered by effect size. **(h)** Genome browser tracks showing CUT&RUN signal for IRF2, IRF4, IRF8, and IgG control, together with scATAC-seq accessibility in TILs at the *Tox* locus. Zoom-in views of selected regions illustrating overlapping IRF-binding and accessible chromatin, supporting direct transcriptional regulation.

**Figure S5.**
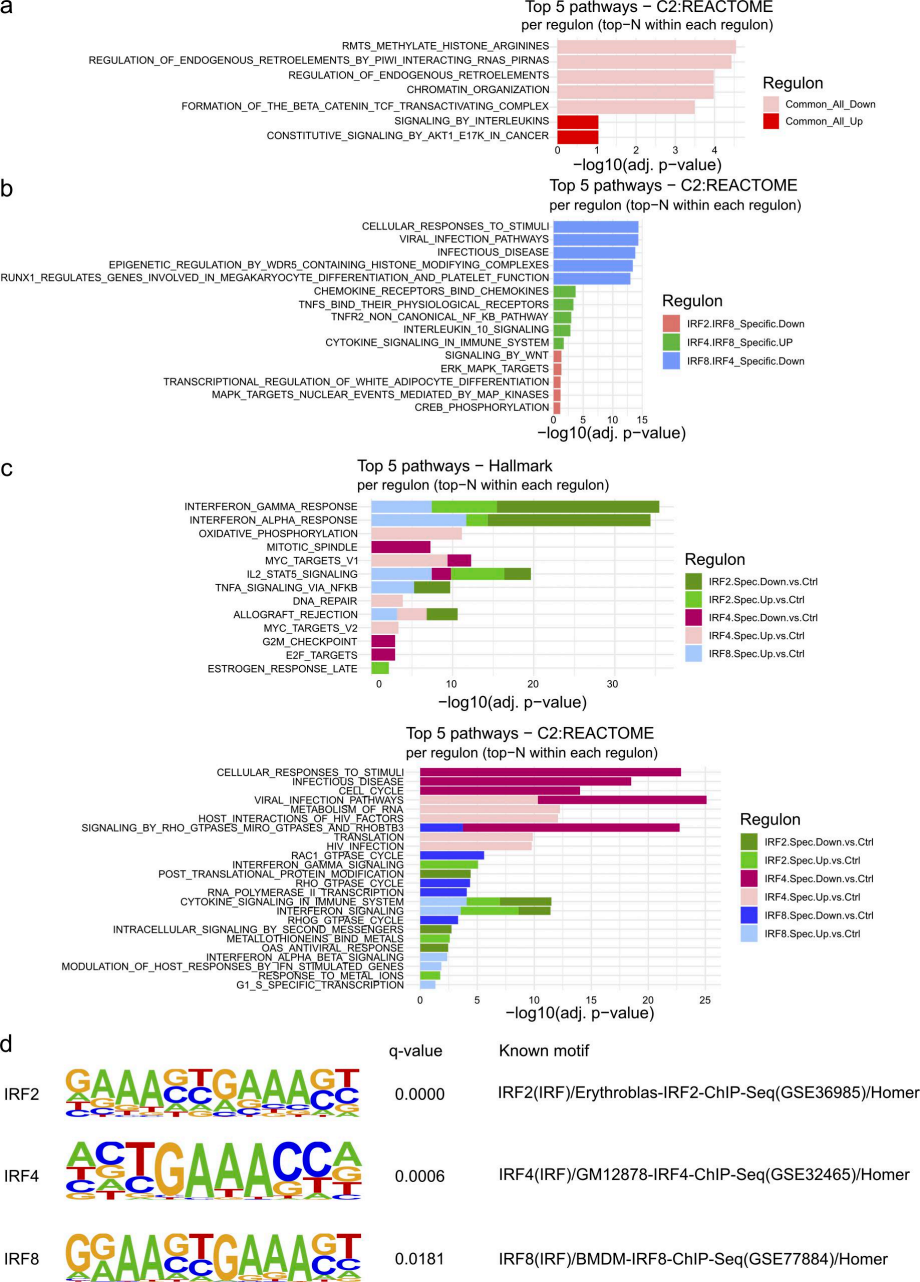
**Overrepresentation analysis reveals enriched pathways upon IRF KO in CD8 TILs. (a and c)** Overrepresentation of pathways from MSigDB Hallmark and MSigDB C2:REACTOME gene sets across up- and downregulated genes. **(a and b)** In a, enriched pathways from C2:REACTOME using up- and downregulated genes common to all KOs and common to IRF2 and IRF8 KO and to IRF4 and IRF8 KO in b are shown. **(c)** In c, enriched pathways using up- and downregulated genes specific to IRF2, IRF4, and IRF8 KO are shown for Hallmark (top) and C2:REACTOME (bottom). **(d)** Homer known motif enrichment analysis done for CUT&RUN data for quality purpose for each of the IRF target, showing significant target enrichment.

We then focused on the differentially expressed genes (DEGs) in the various KO (Table S1 and Table S2). In IRF8 KO, we confirmed our functional data with an increase in mRNA encoding in *Gzmb* and *Ifng* while *Tox* level was decreased (Fig. 8 a). More broadly, these DEG were common to IRF4/IRF8 KO and IRF2/IRF8 KO, while a smaller fraction was shared by the three KOs or by IRF4/IRF2 (Fig. 8, a and b). Among the genes upregulated in IRF4/IRF8 KO, we found *Xcl1*, *Crtam*, *Gzma*, *Tnf*, *Ccl1*, *Ccl3*, *Ccl4*, *Ccl5*, and *Id3*, while *Prf1*, *Gzmf*, *Gzmc*, *Ramp1*, *Isg15*, and *Isg20* were upregulated in IRF2/IRF8 KO cells, suggesting a stronger activation status for the three KOs. The exhaustion-associated genes *Tox*, *Arid1a*, *Arid1b*, and *Ptprj* and the pro-survival gene *Bcl2* were downregulated in all IRF KOs, while *Themis* and *Ikzf2* were specifically downregulated in IRF4/IRF8 KOs. While the overlap of DEGs among the IRF2, IRF4, and IRF8 KO groups was relatively modest (Fig. 8 b), Principal component analysis (PCA) revealed a high degree of transcriptomic proximity between these samples (Fig. 8, c and d). This suggests that while each TF may regulate a distinct set of primary targets, their loss triggers a convergent global gene expression program. The PCA captures these broad, system-wide shifts—including subthreshold expression changes—that reflect a shared phenotypic trajectory toward cellular exhaustion, which is not fully captured by discrete threshold-based Venn analysis. Shared perturbed transcriptional programs analysis between IRF2, IRF4, and IRF8 KO revealed that the three TFs converge on dampening the expression of genes associated with pathways involved in activation or inflammation in tumor-infiltrating CD8^+^ T cells (Fig. 8 e and Fig. S5 a). IRF2 and IRF8 KO resulted in the upregulation of IFN-α/γ responses, IL2/STAT5 signaling, and the allograft rejection signature, pointing to enhanced inflammatory and cytotoxic activity consistent with a shift toward a more effector-like state. IRF4 and IRF8 KO similarly induced TNFα/NF-κB–driven inflammatory together with increased IL-2-STAT5 signaling and allograft rejection signature, again reflecting stronger functional activation. At the same time, both IRF2/IRF8 KO and IRF4/IRF8 KO pairs showed downregulation of pathways linked to cellular homeostasis and persistence, including protein secretion, complement, oxidative phosphorylation, and proliferative programs, suggesting a dual role in establishing exhaustion together with promoting survival (Fig. 8 f and Fig. S5, a–c). Pathways enriched based on each IRF KO-specific up- and downregulated genes were mostly associated to the ones already identified using common DEGs, strengthening our findings (Fig. S5 c).

To further examine the role of IRFs in regulating exhaustion-associated gene expression, we performed CUT&RUN-seq assays for IRF2, IRF4, and IRF8 using CD8^+^ T cells chronically stimulated *in vitro*, as described above. Integration of scRNA-seq and CUT&RUN-seq data identified a list of eight genes (Fig. 8 g) consistently regulated across IRF2, IRF4, and IRF8 perturbations and supported by IRF binding. Genome browser visualization confirmed IRF binding to multiple regions in the *Tox* gene, which coincided with accessible chromatin regions in TILs, supporting the direct regulation of *Tox* by IRF (Fig. 8 h). IRF-bound genes included both upregulated and downregulated genes, indicating that IRFs exert context-dependent regulatory effects rather than uniform activation or repression (Fig. 8 g and Fig. S5 d).

Taken together, these patterns indicate that IRF2, IRF4, and IRF8 converge by promoting exhaustion at least in part through direct regulation of TOX regulation in TILs. KO of the different IRFs similarly promoted a more activated and less exhausted phenotype, underscoring their convergent roles in shaping the exhausted state in TILs.

## Discussion

Despite recent advances, the molecular network governing CD8^+^ T cell differentiation in the context of persistent antigen exposure, i.e., in cancer and chronic infection, is not fully elucidated yet. The IRF family is raising interest for its emerging role in regulating CD8^+^ T cell differentiation and more specifically exhaustion. We established the role of IRF8 as a positive regulator of CD8^+^ T cell exhaustion specifically in a tumor context. Although its expression is TCR-dependent, it was expressed in CD8^+^ TILs but not in virus-specific CD8^+^ T cell during chronic infection. Accessibility of the *Irf8* locus was decreased during chronic infection, with a negative regulatory role for type I IFN, hallmark of chronic infections. CRISPR/Cas9-mediated deletion of IRF8 in tumor-specific CD8^+^ T cells resulted in better tumor control capacity in the setting of ACT in two different tumor models. Overexpressing IRF8 in CD8^+^ T cells increased exhaustion features by increasing TOX expression and limiting cytokine production. Deletion of IRF2, IRF4, or IRF8 in CD8^+^ TILs ameliorated their effector functions in a similar fashion by acting on redundant pathways controlling CD8^+^ T cell differentiation, providing a broader picture of the role of this TF family in exhaustion.

While the function of IRF8 in many immune compartments is well described, its role in CD8^+^ T cells is less clear. Miyagawa et al. (2012) showed that IRF8 is induced by integration of TCR and γc-cytokines in settings of suboptimal antigen stimulation. In their experimental model, IRF8 KO CD8^+^ T cells issued form global *Irf8*^*−/−*^ mice resulted in impaired maturation of CD8^+^ T cells into effector phenotype. Conversely, in Sun et al. (2016), the authors show using CD4-CRE *Irf8*^*fl/fl*^ that targeted IRF8 deletion resulted in more robust immune responses against herpes simplex virus infection. IRF8 KO CD8^+^ cells showed stronger proliferation and higher effector function capacity, measured by IFNγ level assessment, suggesting a role for IRF8 in limiting activation and expansion of CD8^+^ T cells. These seemingly contrasting results underscore the importance of selecting appropriate experimental models to address research questions, as specificity of gene ablation might heavily impact functional outcomes. A more recent publication identified IRF8 as an important factor in reorganization of the spatial proximity between enhancer and promoter regions of exhaustion-associated genes (Li et al., 2025). Li et al. show using a CD8-CRE *Irf8*^*fl/fl*^ experimental mouse model that targeted IRF8 KO in CD8^+^ T cells disrupted differentiation on T_PEX_ into T_EX_, leading to heavily impaired cytokine production, resulting in impaired tumor control capacity. We believe that a major difference explaining the opposing findings in Li et al. (2025) and our work relies on the timing of IRF8 deletion. IRF8 is strongly upregulated shortly after activation, which might indicate that it could exert essential functions during early phases of activation. Using a CRISPR/Cas9-mediated approach on preactivated cells, we believe that we could circumvent these potential confounding effects due to early depletion and better assess IRF8 function in the setting of persistent antigen stimulation, such as in the tumor and during chronic infection. This underscores the need for further investigation of the role of IRF8 in CD8^+^ T cells during early steps of activation.

We found that IRF8 is highly expressed in exhausted CD8^+^ T cells in the tumor but not during chronic infection. As previously reported (Miyagawa et al., 2012; Nelson et al., 1996), we confirmed that IRF8 expression in CD8^+^ T cells is driven by TCR engagement, similarly to its homolog IRF4 (Man et al., 2013). The low expression of IRF8 during chronic infection is interesting and underscores substantial differences between the two conditions, due to inherently distinct microenvironments. We observed clear differences between tumor-specific and virus-specific CD8^+^ T cells at the epigenetic level, providing an explanation to the DE of IRF8 in the two conditions. Chromatin was less accessible at the *Irf8* locus during chronic infection and the H3K27ac modification was lower compared with CD8^+^ TILs, confirming that it is transcriptionally less active. We propose here a role for type I IFN, hallmark of chronic infections (Teijaro et al., 2013; Wilson et al., 2013), as repressor of IRF8 expression during chronic infection. In our experimental models, addition of IFNβ *in vitro* or induction of type I IFN signaling *in vivo* in tumor were able to dampen IRF8 levels in exhausted CD8^+^ T cells. Blocking IFNAR1 in LCMV cl13-infected mice showed opposite effects depending on the timing of treatment. While late blockade during the chronic phase resulted in enhanced IRF8 expression, early blockade during the acute phase of infection decreased IRF8 levels. We think that although our results support the notion that type I IFN is playing a role in altering IRF8 expression in CD8^+^ T cells during chronic infection, additional mechanisms are in place for the DE observed in cancer and chronic infection, and that the biology behind type I IFN signaling in CD8^+^ T cells during chronic infection requires further investigation.

We show here that IRF8 plays a central role in regulating CD8^+^ T cell exhaustion by promoting TOX expression and restricting effector molecules production in tumor-specific CD8^+^ T cells. The comparison of IRF8 KO with IRF2 and IRF4 KO in CD8^+^ T cells allowed us to demonstrate the convergence of these central IRF family members in the regulation of T cell differentiation in the tumor. IRF2 was reported to induce exhaustion in CD8^+^ TILs as a negative feedback on persistent IFN signaling (Lukhele et al., 2022). IRF4, closer to IRF8, has first been described as a TCR-responsive TF driving CD8^+^ T cell exhaustion in LCMV cl13 setting (Man et al., 2017) and tumors (Yan et al., 2023). A subsequent study showed instead that IRF4 was cooperating with its transcriptional partner BATF, when this last was overexpressed, to counter exhaustion in CAR T cells, rewiring the transcriptional outcome as result of redistribution of IRF4 among its target sites in chromatin (Seo et al., 2021). These different reports are issued from different experimental approaches, rendering it difficult to comprehensively integrate the roles of the different TFs. With our approach, we were able to directly compare the cell-intrinsic effect of IRF8 deletion in an unbiased manner by directly comparing IRF8 KO with control TILs issued from the same TME. Moreover, using the same experimental setting, we could directly compare the effects of IRF8 KO with those of IRF2 and IRF4 KO, putting our findings in context of the currently published literature. The three IRFs studied here converge, though by different means, on dampening activation modules and upregulating exhaustion programs in CD8^+^ TILs, unraveling a previously unappreciated redundancy of the IRF family in orchestrating exhaustion. A further point supporting this is the upregulation of the IL2/STAT5 signaling pathway in the IRF2/IRF8 and IRF4/IRF8 KO pairs, as STAT5 has been described to induce a program opposing TOX-driven exhaustion (Beltra et al., 2023). The closer distance between IRF4 and IRF8 KO observed in the PCA analysis and in the shared DEGs suggest a higher degree of redundancy, as already described in B lymphocytes (Shukla and Lu, 2014). Interestingly, a recent publication screening the impact of gene KO in CAR T cells found no advantage to KO IRF2 or IRF4 in their system (Knudsen et al., 2025). It would be interesting to study whether a KO of IRF8, that displayed a better effect on both effector functions and survival/accumulation in our system, has a better effect in CAR T cells engineering too.

The lack of effect of IRF8 KO during chronic infection could also be attributed to the functional redundancy of the various IRF, including IRF2 and IRF4, that may take over IRF8 function. Interestingly, IRF7 has been shown to play an important role in the regulation of CD8^+^ T cell exhaustion during LCMV cl13 infection (Kasmani et al., 2023). IRF7 is barely expressed in exhausted TILs, while its level is much higher in exhausted T cells obtained after chronic infection (Fig. 1 c and Fig. 2 a). Additionally, IRF7 is positively regulated by type I IFN (Fig. 4, g and j), explaining potentially the mutual expression of IRF7 and IRF8 and making of IRF7 a good candidate to take over IRF8 functions during chronic infection.

Altogether, our results established a pivotal role for IRF8 in the context of tumor-induced CD8^+^ T cell exhaustion, but not during chronic infection. The observed functional redundancy of various IRF members during T cell exhaustion underscores the complexity of regulatory networks defining CD8^+^ T cell differentiation trajectories. We confirmed that IRF8 is also expressed human CD8^+^ TILs. Although we did not address the function of IRF8 in human CD8^+^ T cells, our data provide evidence for an application in a human setting. Indeed, a recent study investigating the phenotype of CAR T cells in chronic lymphocytic leukemia found that IRF8 levels were highly enriched in the pre-infusion CAR T cells product from nonresponders compared with complete responders (Fraietta et al., 2018). Moreover, another study found highly reduced TF-binding sites of several IRF members, including IRF8, in open chromatin of CAR T cells with long persistence compared with short persistence, correlated with clinical response, in acute lymphocytic leukemia patients (Bai et al., 2024). The elucidation of mechanistic processes during exhaustion in cancer could unravel new therapeutical opportunities to better harness immunotherapies, for instance, through specific inhibitors or via genetic engineering of IRF8 in tumor-specific CD8^+^ T cells.

## Materials and methods

### Mouse and human samples

Human tumor-infiltrating T cells were obtained from patients treated at the Department of Oncology, University Hospital (CHUV), Lausanne, Switzerland, based on the patient’s informed consent, in the framework of clinical studies (NCT01308294, NCT00112242, NCT00112229, NCT00112216, NCT00002669, LUD 96-010, and PB_2016-02178 [188/12] approved by the Commission cantonale d’éthique de la recherche sur l’être humain). Frozen human samples were analyzed by flow cytometry analysis to assess IRF8 protein levels on a single-cell resolution.

The experiments conducted in this research project comply with Swiss ethical regulations. Experiments with animals were performed in compliance with the University of Lausanne’s internal regulations and approved by the “Service de la consummation et des affaires vétérinaires” (authorization VD3593). All mice used for the experiments were housed in a specific-pathogen free facility of the University of Lausanne. To avoid sex bias, male and female mice were used in the whole project.

C57BL/6 (B6) (CD45.2^+^) mice were obtained from Charles River. CD45.1 congenic B6 mice were bred locally. B6 P14 TCR-transgenic mice (line 237), expressing a TCR specific for the LCMV gp33–41 epitope (gp33) in the context of H-2D^b^ (P14 T cells), were initially provided by H.P. Pircher (Institute for Immunology, Medical Center, University of Freiburg, Freiburg, Germany) (CD45.2^+^) (Pircher et al., 1989). CD45.1 and CD45.1.2 P14 CD4-CRE STOP^fl/fl^-Cas9-eGFP (P14-Cas9) were used to induce targeted KO of selected genes. B6 Rosa26-Cas9-eGFP knock-in mice (B6-Cas9) mice were used as host mice for transfer experiments of Cas9-expressing T cells to avoid rejection. Cas9 strains were kindly provided by P.C. Ho (University of Lausanne, Lausanne, Switzerland [Franco et al., 2023]). OT-I CD45.1 and *Rag1*^*−*^ mice were bred in-house. CD45.1 Vβ5 mice were used as hosts for experiments with infection and were kindly provided by W. Held.

### Cell culture and cell lines

For cell culture we used RPMI 1640 GlutaMAX-I (Gibco) and DMEM GlutaMAX-I (Gibco) media. Complete RPMI was supplemented with 10% heat-inactivated FBS (Gibco), 100 U ml^−1^ penicillin-streptomycin (Gibco), 10 mM Hepes (Gibco), 1 mM sodium pyruvate (Gibco), and 50 μM 2-mercaptoethanol (Gibco). Complete DMEM was supplemented with 10% heat-inactivated FBS and 100 U ml^−1^ penicillin-streptomycin. Primary murine CD8 T cells were cultured in complete RPMI. Frozen human melanoma metastasis samples were thawed and cultured ON with complete RPMI. B16F10, LLC1, EL-4, and MC38 cell lines were cultured in complete DMEM. B16-gp33 and B16-OVA were cultured in complete DMEM with 100 μg ml^−1^ G418 (Calbiochem). PlatinumE (PlatE) cells were cultured in complete DMEM with 10 μg ml^−1^ blasticidin (Invivogen) and 1 μg ml^−1^ puromycin (Invivogen).

### Vectors, retrovirus production

The various sgRNA or scrambled controls were inserted into a PQG-Thy1.1 retroviral vector (Table S4), kindly shared by P. Reichenbach (Melita Irving’s lab, University of Lausanne, Lausanne, Switzerland). The IRF8-coding sequence used for OE experiments was purchased from Addgene (plasmid number 102872) and was inserted into pMSGV retroviral vector. The sequence was cloned using In-Fusion Cloning kit (Takara Bio) after the Thy1.1 transduction marker, separated by a self-cleaving P2A sequence. As control for OE experiments, a pMSGV vector containing only Thy1.1 was used.

For retroviral production, 7 × 10^6^ PlatE cells were seeded into a T75 flask ON before transfection. PlatE cells were then transfected using a mix of Lipofectamine 2000 (Invitrogen) with a total of 30 μg of DNA (7.5 μg pCL-Eco and 22.5 μg of PQG-sgRNA-Thy1.1 or pMSGV-Thy1.1) in pure DMEM. The culture medium was replaced after 24 h with complete RPMI. Retroviral supernatants were recovered after 48 h, filtered, snap-frozen, and stored at −80°C.

### CD8^+^ T cell transduction for KO and OE experiments

As efficient retroviral vector integration requires active cell proliferation, CD8^+^ T cells were transduced 24 h after activation with 1 μg ml^−1^ GP33 peptide (KAVYNFATC) and 20 U ml^−1^ recombinant human IL-2 (Proleukin aldesleukin) in 1.5 ml complete RPMI. For the transduction, 1 ml of culture medium was removed and replaced with 1 ml of retroviral supernatant supplemented with 10 μg ml^−1^ polybrene infection/transfection reagent (Sigma-Aldrich). Cells were spun for 60 min at 1,800 r.p.m. at 30°C and incubated 2 h at 37°C. 1 ml of medium was then removed and replaced by the initially removed medium.

### RNA extraction for RT-qPCR

For validation of IRF2, IRF4, and IRF8 KO, transduced KO and control CD90.1^+^ P14 CD8 Cas9 T cells were FACS sorted 48 h after retroviral transduction. RNA was extracted with the RNeasy Plus Micro Kit (Qiagen) following the manufacturer’s recommendations. Reverse transcription was done with the High-capacity cDNA Reverse Transcription kit (Applied Biosystems), and qPCR was done with the KAPA SYBR Fast qPCR Master Mix (2x) Kit (Sigma-Aldrich). Primers are listed in Table S4. Amplification was done in 96-well plates on a QuantStudio 6 Pro Real-Time PCR systems machine (Applied Biosystems), and data were analyzed with the Design & Analysis 2 software.

### Tumor experiments

Tumor cells were resuspended in PBS before s.c. injections into the flank of mice. 0.5 × 10^6^ cells for B16F10, B16-gp33, and B16-OVA, and 1 × 10^6^ cells for MC38, LLC1, and EL-4 were injected in 50 μl PBS volume. Orthotopic MIBC, used to assess IRF8 expression in exhausted CD8^+^ T cells, was induced though injection of a CRE-encoding adenovirus in the bladder of *p53*^*fl/fl*^*/pten*^*fl/fl*^ mice, as described elsewhere (Leblond et al., 2020). Analysis was performed 8 wk after tumor initiation. For naive CD8^+^ T cell transfer experiments, CD8^+^ T cells were enriched by negative selection following the manufacturer’s instruction (STEMCELL) and intravenously transferred at 2 × 10^6^ cells/mouse. For preactivated CD8^+^ T cell transfer experiments, LN cell suspensions were cultured in complete RPMI + 1 μg ml^−1^ GP33 peptide (KAVYNFATC) or 1 μg ml^−1^ OVA peptide (SIINFEKL) with 20 U ml^−1^ recombinant human IL-2 (Proleukin aldesleukin) for 48 h before intravenous injection in mice. For KO and OE experiments, retroviral transduction was performed at 24 h after activation. Cells were counted and mixed in a 1:1 ratio, with a total of 2 × 10^6^ CD8^+^ T cells injected per mouse (e.g., 1 × 10^6^ P14 control + 1 × 10^6^ P14 IRF8 KO CD8^+^ T cells). 7 days after T cell transfer mice were sacrificed and tumors, draining and non-draining LN were collected and passed through 70-μm cell strainer. Lymphocytes were enriched from tumors using a 40/70 Percoll gradient (VWR 17-0891). For tumor growth experiments, 4 × 10^6^ CD8^+^ T cells after 48 h *in vitro* activation were injected intravenously 7 days after tumor engraftment. Tumor volumes were assessed with a caliper and calculated using the following formula: volume (mm^3^) = length (mm) × width (mm) × height (mm). Endpoint for tumor growth experiments was 1 cm^3^ of tumor volume in compliance with the animal experimentation license.

### LCMV cl.13 and Arm infection experiments

Vβ5 CD45.1 mice were infected by intravenous injection with 2 × 10^6^ PFUs of LCMV cl13 1 day after ACT of 1 × 10^3^ naive P14 CD45.2 CD8^+^ T cells for chronic infection. CD45.1^+^CD45.2^+^ B6 mice were injected with 2 × 10^5^ PFUs of LCMV 53b Arm strain 1 day after ACT of 1 × 10^4^ naive P14 CD45.2 CD8^+^ T cells for acute infection. For IRF8 KO and IRF8 OE co-transfer experiments, transduced CD8^+^ T cells were FACS sorted based on CD90.1 expression and injected in a ratio KO/OE: Control of 1:1 at 4 × 10^4^ CD8^+^ T cells per mouse on the same day of infection. On the readout day, spleens were recovered, passed through cell strainers, and incubated 5 min with RBC lysis buffer before further processing. Livers were passed through cell strainers, and lymphocytes were enriched with a 40/70 Percoll gradient. For IFNAR1 blockade, mice were injected i.p. with 1 mg of anti-IFNAR1 (MAR1-5A3; BioXCell) (1 mg) or 1 mg of isotype control Ab (mouse IgG1, MOPC-21, BioXCell) at day 3, days 10 and 15, or day 22 after infection, as indicated in the Fig. 4.

### *In vitro* chronic TCR stimulation protocol

CD8^+^ T cells were enriched by negative selection following the manufacturer’s protocol (STEMCELL). 0.4 × 10^6^ CD8^+^ T cells per well were plated in complete RPMI in a 24-well plate, previously coated with anti-mouse CD3 antibodies (clone 145-2C11; BioLegend) at 5 μg ml^−1^, and activated for 72 h with addition of soluble anti-mouse CD28 (clone 37.51; BioLegend) at 1 μg ml^−1^ and 20 U ml^−1^ IL-2. Every 48 h, cells were plated to new anti-CD3–coated 24-well plate with 10 U ml^−1^ IL-2 at 1 × 10^6^ cells per well. This step was repeated two additional times for a total of three restimulations after initial activation. For the resting condition, after the initial, activation cells were plated in complete RPMI supplemented with 5 ng ml^−1^ IL-7 (PeproTech) and 5 ng ml^−1^ IL-15 (PeproTech), and cytokines were refreshed every 2 days.

### Flow cytometry

Fluorescent antibody staining was performed for all experiments with the same protocol. Cells were incubated with anti-mouse CD16/32 (101320; BioLegend) in FACS buffer (PBS with 2% FBS, 5 mM EDTA, and 0.2% Na azide) for 15 min. Extracellular staining was then performed for 30 min at 4°C with an antibody mix in FACS buffer. Cells were washed twice with PBS and subsequently stained for viability with Aqua Vivid (L34966; Invitrogen) or Zombie NIR (423105; BioLegend) for 15 min at 4°C. Cells were then analyzed directly on a cytometer or fixed and permeabilized for intracellular/intranuclear staining for 20 min at 4°C using the FoxP3 staining kit (00-5523-00; Invitrogen). After one wash in permeabilization buffer, cells were incubated for 1 h at 4°C for intracellular/intranuclear staining. For cytokine staining, P14 CD8^+^ T cells were restimulated for 5 h at 37°C in presence of GP33 peptide in addition to GolgiStop (554724; BD). The complete list of antibodies used can be found in Table S3.

### Swiss Portal for Immune Cell Analysis (SPICA) atlas

The expression profile figures of IRF genes from Fig. 1, c and g were created based on the publicly available SPICA atlas for tumor-infiltrating T cells and virus-specific CD8 T cells atlases (Andreatta et al., 2022). This collection comprises a highly curated and annotated collection of cancer and infection transcriptomic studies describing immune cell states at high resolution.

### Regulon analysis

The input consisted of a publicly available, carefully curated compendium of scRNA-seq profiles from mouse CD8^+^ TILs (Andreatta et al., 2021). The collection covers five well-defined, naturally occurring CD8^+^ states with distinct transcriptional signatures: CD8_NaiveLike, CD8_EffectorMemory, CD8_EarlyActiv, CD8_Tpex, and CD8_Tex. Regulatory programs (regulons) were inferred with the SCENIC workflow (Aibar et al., 2017) (https://scenic.aertslab.org). To capture regulatory events specific to individual subpopulations, the dataset was partitioned by T cell state, and each subset was processed independently. SCENIC proceeds in three sequential stages—grnBoost2, RcisTarget, and AUCell—which we used as follows: Step 1: Co-expression modules (grnBoost2). Co-expression modules were derived using grnBoost2—a fast implementation related to GENIE3 (Huynh-Thu et al., 2010)—via the SCENIC R package. We invoked runSCENIC_1_coexNetwork2modules with nTopTfs = 50 and nTopTargets = 5 to aggregate targets into preliminary (raw) putative regulons. Step 2: Regulon/GRN refinement (RcisTarget). Modules were pruned to remove indirect targets by motif enrichment analysis with RcisTarget (Herrmann et al., 2012; Imrichová et al., 2015) and cis-regulatory motif resources. Specifically, we used the cisTarget ranking databases mm9-500bp-upstream-7species.mc9nr.feather and mm9-tss-centered-10kb-7species.mc9nr.feather, together with the motif table motifs-v9-nr.mgi-m0.001-o0.0.tbl. The motif database provides scores for motif–gene pairs, yielding per-TF motif–gene rankings. For each candidate TF target set, motif enrichment was quantified as the area under the recovery curve on these rankings (Aibar et al., 2017). When a TF’s motif was significantly enriched, a regulon was called by retaining target genes with high motif–gene scores; these curated TF-target edges formed the GRN.

### Discriminant analysis

We applied Orthogonal Partial Least Squares Discriminant Analysis (OPLS-DA), a multivariate statistical approach that leverages predefined class labels to reduce variability within each class and enhance separation between classes (Thévenot et al., 2015). The method produces a predictive component (p1), which represents a data projection that optimally distinguishes elements (cells) belonging to the two predefined groups (T cell states). Each feature (such as a gene or regulon) contributes to this predictive component, quantified by a value referred to as weightStarMN. To facilitate comparison, these values are rescaled to a range between 0 and 1 while retaining their sign, yielding the discriminant score for each feature. Within a given OPLS-DA model, these scores allow features to be ranked according to their relative importance in distinguishing between the classes. The OPLS-DA was performed using the ropls R packageREF. The input data for OPLS-DA were mean centered and divided by the SD to ensure proper normalization.

### Multiplexed scRNA-seq experiments

The scRNA-seq experiment was performed on *ex vivo* FACS-sorted CD8^+^ TILs issued from a co-transfer experiment. Mice were engrafted with B16-gp33 tumors, followed by a co-transfer of retrovirally transduced P14 control together with P14 IRF(2, 4, or 8) KO CD8^+^ T cells at day 7. 7 days after adoptive CD8^+^ T cell transfer, mice were sacrificed, and tumors were passed through a 70-μm cell strainer. CD8^+^ T cells were enriched using a positive CD8^+^ TILs selection (Miltenyi), following the manufacturer’s instructions. After CD8^+^ TILs enrichment, cells were stained for FACS sorting as described above (viability marker, CD8-biotin, CD45.1, CD45.2, and CD90.1). In a second step, cells were incubated with TotalSeq hashtagging streptavidin-APC antibodies (BioLegend) for multiplexing. Control and IRF KO cells issued from the same tumor were thus labeled with the same hashtagging sequence, allowing for a paired analysis of cells exposed to the same TME. The samples were pooled and FACS sorted based on viability and the expression of CD8a, CD90.1, and CD45.1/CD45.1.2 to separate P14 control from P14 IRF KO cells. Sequencing was performed on two lanes of an Element Aviti System device at the Lausanne Genomic Technologies Facility (https://wp.unil.ch/gtf/).

### scRNA-seq data processing and quality control

Raw scRNA-seq data were obtained from multiple experimental batches of IRF2, IRF4, and IRF8 control and KO samples. CellRanger-processed gene expression and multiplexing count matrices were imported into R (version 4.x) using Seurat (version 5) and related packages (SeuratWrappers, STACAS, scRepertoire, scGate, UCell, ProjecTILs, scIntegrationMetrics, and batchelor). For each dataset, Seurat objects were generated from the gene expression matrices and multiplexing capture assays, with sample- and condition-specific metadata annotations (condition, sample, batch, and Ctrl/KO). Gene expression counts were normalized per dataset prior to merging. To identify and exclude confounding cellular states, transcriptional signatures corresponding to cell cycle (G1/S, G2/M), IFN-stimulated genes (ISGs), and heat shock proteins (HSP) were derived from the SignatuR database and custom gene sets. UCell scores were computed per cell for these signatures across all datasets. All Seurat objects were merged into a single dataset and subjected to quality control filtering. Quality metrics included the number of detected genes (nFeature_RNA), UMI counts (nCount_RNA), and the proportion of reads mapping to ribosomal (Rp[sl]) and mitochondrial (mt-) genes. Cells were retained if they satisfied the following thresholds: nFeature_RNA: 800–4,900, nCount_RNA: 1,300–24,000, percent.ribo <50%, and percent.mito <10%. After filtering, the merged dataset was saved as a Seurat object for downstream analysis. Analyses were performed in R using Seurat (object handling, normalization, and HVG selection), PCAtools (dimensionality reduction, scree diagnostics, and biplots), ggplot2 (visualization), tidyverse/dplyr (data wrangling), Matrix, and ProjecTILs (heatmap utility). The SignatuR resource provided gene signatures. The in-house package ScPlusPlus was installed from GitHub and loaded for utilities. Default settings were used unless otherwise specified. A previously generated Seurat object containing merged scRNA-seq profiles from IRF2/IRF4/IRF8 control and KO conditions was read from disk. Sample-level metadata included condition, sample, batch, and previously computed signature scores (e.g., cycling). Cells were binarized into Prolif versus NoProlif based on the cycling signature score threshold (cycling ≥ 0.1 → Prolif; otherwise NoProlif). Counts per condition and per sample were tabulated to quantify group sizes. For both condition and sample strata, cell counts were aggregated by Prolif class, and two normalizations were computed: (1) normSize.Condition = cells in class/total cells within each condition (or sample) and (2) normSize.Cluster = cells in class/total cells within each Prolif category.

Class composition within each Prolif group was further adjusted by dividing normSize.Condition by the sum across classes for that group (clustComposition). Bar plots of normalized frequencies were generated and exported to PDF. For each batch (analyzed separately for batch1 and batch2), cells were subset to the given batch label and processed as follows: (1) Normalization: Seurat LogNormalize with scale.factor = 10,000. (2) Feature selection: FindVariableFeatures with selection.method = “dispersion” and nfeatures = 5,000. (3) Feature blacklist and pruning: High-variance features were filtered to exclude genes associated with confounding programs and technical artifacts. Gene sets included: Cell cycle G1/S and G2/M (from SignatuR), ISG genes (custom list: *Isg15*, *Ifit3*, *Ifit1*, *Rsad2*, *Usp18*, *Isg20*, *Bst2*, *Phf11b*, *Zbp1*, *Stat1*, *Slfn5*, *Samhd1*, *Iigp1*, and *Ifit3b*), heat shock genes (from SignatuR HeatShock), mitochondrial (prefix mt-), ribosomal (regex ^Rp[ls]), TCR (regex Tr[abgd][vjd]), and a custom blacklist (external resource). The final HVG set was defined as the initial 5,000 HVGs minus all blacklisted genes. Using ProjecTILs::celltype.heatmap with assay = “RNA” and the pruned HVG set, a sample-by-gene matrix (clustered by genes and samples) was computed and returned. Zero-sum columns (genes) were removed. Sample row names were parsed to extract state and batch identifiers; a metadata table was built with fields subset (=State), source (=Condition parsed from sample names), and a binary Ct_KO label (ctrl versus KOs). For each batch, the top 2,000 HVGs present in the summary matrix were retained, and the matrix was transposed to genes × samples for PCA. PCA was performed with PCAtools::pca using removeVar = 0.1 (removing the lowest 10% variance features). The number of informative components was assessed with parallelPCA (Horn’s test) and elbow detection from the variance profile. PCA biplots were generated with PCAtools::biplot, coloring samples by source with a predefined color key and encircling groups. For batch2, a single outlier sample (I4.KO_r3) was removed prior to PCA. Normalized composition bar plots and PCA diagnostics (scree plots with Horn’s and elbow references, PCA biplots) were produced with ggplot2 and PCAtools.

### Data integration

All analyses were performed in R using Seurat (object handling, normalization, QC, and clustering), STACAS (batch integration), UCell (signature scoring), UMAP via Seurat, ProjecTILs (utilities), ggplot2/patchwork (visualization), dplyr/tidyr/tidyverse (wrangling), Matrix, gridExtra, and scIntegrationMetrics. Gene programs were retrieved from SignatuR. Unless otherwise noted, default parameters were used, and Seurat objects were handled in assay version “v3.” A previously curated Seurat object containing merged scRNA-seq profiles from IRF2/IRF4/IRF8 control and KO conditions was loaded and split by the batch metadata. Mouse gene annotations (EnsemblGeneTable.Mm) were used to standardize gene symbols, and each cell’s barcode was stored in metadata. Objects were slimmed to the RNA assay and log-normalized with Seurat’s NormalizeData (scale.factor = 10,000). Three confounding programs were defined: Cycling = union of G1/S and G2/M (SignatuR), ISG = curated ISG set (*Isg15*, *Ifit3*, *Rsad2*, *Stat1*, *Samhd1*, etc.), HSP = heat-shock program (SignatuR). Per-cell UCell scores were computed within each batch. Cells with elevated stress or IFN signatures were excluded (ISG < 0.25 and HSP < 0.6). Across batches, quality control thresholds were derived from the 2nd–98th percentiles of nCount_RNA (UMIs) and nFeature_RNA (genes), and only cells within these ranges were retained. Batch-specific HVGs were identified with STACAS::FindVariableFeatures.STACAS (requesting twice the target number, with nfeatures = 1,000 downstream). HVGs were pruned against comprehensive blocklists consisting of cell cycle, ISG, and HSP programs (from SignatuR), mitochondrial genes (^mt-), ribosomal genes (^Rp[ls]), and TCR segments (Tr[abgd][vjd]), and an external blacklist plus specific entries (*Trbv12-1*, *Trbv13-2, Trgv2*, *Trbc1*, and *Seq1*). For each batch, pruned HVGs were defined as setdiff(HVG, blocklists); pre- and post-filter HVG counts were recorded. A final cross-batch HVG panel was selected with SelectIntegrationFeatures (nfeatures = 1,000). Objects were integrated using STACAS::Run.STACAS (dims = 1:30, anchor.features = HVG panel), followed by UMAP embedding (dims = 1:30, seed = 123). Marker expression (e.g., *Tox*, *Pdcd1*, *Gzmb*, *Cd69*, *Tcf7*, *Ifng*, and *Cx3cr1*) was inspected on the RNA assay. Cells were clustered in the integrated space via shared nearest neighbor graphs (FindNeighbors, k.param = 20) and Louvain community detection (FindClusters, algorithm = 1) across multiple resolutions (0.2–0.4). Resolution 0.2 was selected. Cluster expression profiles were visualized by violin plots across markers spanning progenitor, exhaustion, effector, and proliferative programs (*Lef1*, *Tcf7*, *Pdcd1*, *Tox*, *Gzmb*, *Ifng*, *Zeb2*, *Klrg1*, *Mki67*, etc.). Clusters were then manually annotated and stored in the Seurat. Annotation field (e.g., *C1_Tex*, *C2_Tex_Cycling*, *C3_Tpex*, and *C5_ToxLow_EffLike*). Cluster abundances were tabulated per condition and sample. For each cluster, relative frequencies were computed as: normSize.Condition = cells in cluster ÷ total cells in that condition/sample, normSize.Cluster = cells in cluster ÷ total cells of that cluster. Cluster composition was further normalized within clusters to give clustComposition. Bar plots of relative cluster frequencies.

### DE and GSEA

Analyses were performed in R using Seurat (object handling), edgeR (pseudobulk DE), fgsea, ScPlusPlus (GSEA helper utilities), tidyverse/dplyr/tidyr (wrangling), Matrix, ggplot2/patchwork (visualization), and ProjecTILs utilities. Seurat objects were handled in assay version v3 unless noted. An integrated Seurat object (object_integrated_ss_full,for.DEA.rds) was loaded and the default assay set to RNA. Cells were annotated by cluster/state in Seurat.Annotation. The genotype comparison was defined per IRFx target as KO versus control (e.g., baseline_target = “IRFxc,” contrast_target = “IRFxko”). Sample strings were parsed from the sample metadata to extract target (IRFx), genotype (C/KO), and replicate_id (e.g., T1, T2). Only balanced replicates (those containing both genotypes for the same IRFx target) were retained for analysis. Gene-level raw counts were aggregated per (replicate_id × genotype) by summing cell counts (sparse matrix row sums), producing a pseudobulk counts matrix. A corresponding sample metadata table recorded replicate and genotype for each pseudobulk column, with genotype factors ordered so that control served as the baseline. Pseudobulk counts were filtered using edgeR’s filterByExpr (blocking on replicate), library sizes normalized with TMM, and gene-wise/BCV dispersions estimated. A paired GLM with design replicate_id + genotype\texttt{∼ replicate\_id + genotype} replicate_id + genotype was fit using glmQLFit, and the genotype coefficient tested with glmQLFTest to obtain moderated quasi-likelihood statistics. Positive logFC indicates higher expression in IRFxKO relative to IRFxc within replicate. Full DE tables were exported as CSV (e.g., deg.IRF4KO_vsCtril_considering_pairing.csv). Genes were ranked by logFC from the edgeR contrast (IRFxKO versus IRFxc). Ranked lists were analyzed with ScPlusPlus::run_msigdbr_gsea_for_ranked_lists (wrapper around fgsea) against MSigDB C7: IMMUNESIGDB gene sets, filtered by the keyword “EFFECTOR_VS_EXHAUSTED.” GSEA parameters included top_*n* = 20, padj_cutoff = 0.1, and dot plots colored by NES and sized by leading edge. Enrichment plots were generated and saved as PDFs for the all-cells analysis and for the Tpex and Tex scopes; GSEA result tables were also written to disk.

### DE visualization and pathway overrepresentation analyses

All analyses were performed in R using Seurat (object handling), EnhancedVolcano (volcano plots), VennDiagram (set overlaps), ggplot2/patchwork (visualization), tidyverse/dplyr/tidyr (data wrangling), matrix, and ScPlusPlus (MSigDB enrichment wrappers). Seurat objects were handled in assay version v3 unless otherwise noted. An integrated Seurat object (object_integrated_ss_full, for.DEA.rds) was loaded for enrichment analyses. DE results from a paired pseudobulk design (KO versus control) were imported from CSV files for IRF2, IRF4, and IRF8. Each DE table contained at least gene, logFC, and FDR and was available at multiple scopes: all cells, Tpex (C3_Tpex), and Tex (C1_Tex). A curated gene list (MarcoList.csv) was used to highlight specific markers in plots. DE tables were visualized with EnhancedVolcano. Plots emphasized canonical CD8 T cell markers (e.g., *Tox*, *Tcf7*, *Pdcd1*, *Gzma/b/c/d/e/f*, *Havcr2*, *Klrg1*, and *Cx3cr1*), the curated list from MarcoList.csv, and the most extreme DE genes (top/bottom by logFC). Nominal thresholds of FDR <0.05 and |logFC| ≥0.3 guided labeling/interpretation, with axis ranges tuned per comparison. Figures were exported as PDFs (e.g., VolcanoPlot_deg.IRF8KO_vsCtril.pdf, plus Tpex and Tex variants). Within Tex comparisons, DE genes were classified as upregulated (logFC > 0.3, FDR < 0.05) or downregulated (logFC less than −0.3, FDR <0.05). From these, multiple contrasts were derived: (1) Target-specific sets: unique to each IRF after subtracting overlap with the other two (e.g., IRF2.Spec.Up, IRF4.Spec.Down). (2) Common sets: genes shared across all three IRFs (Common_All_Up, Common_All_Down). (3) Pair-specific overlaps: shared between two IRFs but absent in the third (e.g., IRF2.IRF4_Specific.Up, IRF8.IRF4_Specific.Down). Venn diagrams (PNG format) visualized overlaps among up or down gene sets, using custom colors and transparency. Pathway enrichment was performed with ScPlusPlus::run_msigdbr_ora_for_regulons, using the integrated object as background. Analyses targeted MSigDB collections C2: REACTOME and H: Hallmark (species = mouse). For C7, terms were filtered with keywords “CD8” and “IFN” in “all” mode. Parameters included top_*n* = 5, padj_cutoff = 0.05–0.10, and plot_style = “bar.” Enrichment bar plots were color-mapped to gene set categories and exported as PDFs (e.g., Immune_BAR.Up_Specific.pdf, Reactome_BAR.Up_SpecificPairs.pdf). All volcano plots, Venn diagrams, and ORA bar plots were saved in the plots/directory. Intermediate and combined gene lists were retained for plotting. Package versions and environment details were recorded via sessionInfo.

### scATAC-seq processing and analysis

All analyses were performed in R version 4.3. Package dependencies included ArchR (core processing), Seurat/Signac (object conversion/visualization), ggplot2 (version 3.4.4; plotting), pheatmap (heatmaps), BSgenome.Mmusculus.UCSC.mm10 (genome), EnsDb.Mmusculus.v79 (gene annotation), and ArchRtoSignac (conversion utilities). Unless otherwise noted, the mouse reference genome was mm10. Computations used 16 threads (addArchRThreads(threads = 16)), and stochastic steps used a fixed seed = 1,234 for reproducibility. Accessible fragment files (three samples: Cl13d8, Cl13d30, naive (GSE199565 [Giles et al., 2022]) and TIL_NFAT5_wt (GSE237711 [Tille et al., 2023]) were provided as fragments.tsv.gz. For each sample, we created Arrow files with: Cell QC thresholds: minTSS = 15, minFrags = 5,000, maxFrags = 50,000, Matrices: genome-wide TileMatrix (500-bp bins) and GeneScoreMatrix (from the mm10 gene annotation defined via addArchRGenome(“mm10”)), Command: createArrowFiles(..., addTileMat = TRUE, addGeneScoreMat = TRUE, force = TRUE). Arrow files were used to instantiate an ArchRProject (copyArrows = TRUE), which was then filtered for doublets. Putative doublets were scored per sample using addDoubletScores with k = 10, knnMethod = “UMAP,” and LSIMethod = 1. Cells flagged as doublets were removed via filterDoublets. We performed iterative LSI on the TileMatrix in two rounds: (1) IterativeLSI (v1), addIterativeLSI(name = “IterativeLSI,” iterations = 4, varFeatures = 10,000, dimsToUse = 1:20, clusterParams = list(resolution = c(0.1,0.2,0.4), sampleCells = 10,000, *n*.start = 10), seed = 1,234, force = TRUE). (2) IterativeLSI (v2), addIterativeLSI(name = “IterativeLSI_2,” iterations = 4, varFeatures = 20,000, dimsToUse = 1:20, clusterParams = list(resolution = c(0.1,0.2,0.4), sampleCells = 10,000, *n*.start = 10), seed = 1,234, force = TRUE). ArchR’s depth-correlated LSI components were automatically handled (default corCutOff). SDs per component were inspected to confirm dimensionality choice. Graph clustering used Seurat’s Louvain/Leiden implementation via ArchR: Round 1: addClusters(reducedDims = “IterativeLSI,” name = “Clusters_1,” resolution = 0.2, seed = 1,234, force = TRUE). Round 2: addClusters(reducedDims = “IterativeLSI_2,” name = “Clusters_2,” dimsToUse = 1:20, resolution = 0.3, seed = 1,234, force = TRUE). Cluster/sample relationships were quantified with confusion matrices and visualized as heatmaps (pheatmap) after row-normalization. UMAP embeddings were computed from the LSI spaces: UMAP_1: addUMAP(reducedDims = “IterativeLSI,” nNeighbors = 30, minDist = 0.5, metric = “cosine,” name = “UMAP_1,” force = TRUE). UMAP_2: addUMAP(reducedDims = “IterativeLSI_2,” dimsToUse = 1:20, nNeighbors = 50, minDist = 0.5, metric = “cosine,” name = “UMAP_2,” force = TRUE). Cells were visualized with plotEmbedding, colored by sample, Clusters_1, Clusters_2, or user-defined meta-annotations (see below). Custom theming was applied for publication figures. Samples were grouped into experimental categories for downstream summaries: Chronic LCMV (Cl13d8, Cl13d30) naive and TILs. A derived factor Infection_vs_TIL (levels: naive, Arm, Cl13, and TILs) and batch labels were added (Cl13/Naive → “Giles_Wherry_NI_2022”; TILs → “Greg_NI_2024”). Pseudobulk replicates were created per Clusters_2 with addGroupCoverages(groupBy = Clusters_2, force = TRUE). Peaks were called using MACS2 discovered by findMacs2(), and a reproducible peak set was generated with: addReproduciblePeakSet(groupBy = Clusters_2, pathToMacs2 = ..., force = TRUE). The resulting peak set (as GRanges) was retrieved via getPeakSet() and saved to disk. A PeakMatrix was then added (addPeakMatrix(force = TRUE)), and available matrices were enumerated for QC. Browser tracks around marker genes were produced with plotBrowserTrack: Tox: ±500 kb window; grouped by Sample and restricted to selected groups (“naive,” “Cl13d8,” “Cl13d30,” and “TILs”), Irf8: ±30 kb window (same grouping approach). Plots were rendered to PDF (plotPDF). Group-level bigWigs were exported with: getGroupBW(groupBy = “Sample,” normMethod = “ReadsInTSS,” tileSize = 100, maxCells = 1,000, and ceiling = 4).

### scATAC-seq, Cut&Tag, and ChIP-seq data visualization

The aforementioned ATAC-sequencing bigWig files and H3K27ac Cut&Tag bigWig files were utilized for epigenetic analysis in selected loci. The H3K27ac data were sourced from the Gene Expression Omnibus (GEO) under accession number GSE235007 (Ma et al., 2025). Additionally, TF-binding regions for mouse CD8^+^ T cells were obtained from the ChIP-Atlas public database (Zou et al., 2022). Genomic tracks were visualized using the Integrative Genomics Viewer (IGV) version 2.16.0 (Kent et al., 2002). H3K27ac signal intensity within specific genomic regions, including the Irf8 and Tox loci, was quantified using the UCSC command-line utility bigWigAverageOverBed.

### CUT&RUN-seq

CD8^+^ T cells from WT B6 mice were enriched by negative selection following the manufacturer’s protocol (STEMCELL) and cultured following the *in vitro* chronic TCR stimulation protocol described above to induce exhaustion. CD8^+^ T cells were then FACS sorted and processed for CUT&RUN-seq following the manufacturer’s instructions (EpiCypher) with the modification of increasing the bead concentration to ensure smaller fragments were not lost during the purification process. Libraries were pooled and subjected to three further rounds of bead purification to exclude the adapter dimers present in the libraries. Libraries were sequenced on a NovaSeq instrument in 150-bp paired-end mode. CUT&RUN sequencing data were aligned to the mouse reference genome (mm10) using Bowtie2 (version 2.4.5), and BAM files were processed using SAMtools (version 1.19). Genome-wide signal tracks were generated using deepTools (version 3.5.5; bamCoverage) with CPM normalization to account for differences in sequencing depth across samples. Peak calling was performed using SEACR (version 1.3) in stringent mode with IgG as control. Reproducible peaks across replicates were identified using BEDTools (version 2.31.1), and peaks were annotated to genes using HOMER (version 4.11). BigWig files were generated using UCSC utilities, including bigWigMerge and bedGraphToBigWig (UCSC tools version 445), and used for downstream visualization. Direct targets were defined by intersecting DEGs with genes associated with CUT&RUN peaks. Shared targets across IRF2, IRF4, and IRF8 were defined as genes differentially expressed in all three perturbations and bound by at least one IRF. Lollipop plots were generated in R (version 4.4.1), where the x-axis represents log2 fold change and point size indicates –log10(FDR). For shared targets, log2 fold change values from the IRF8 KO condition were used. Genome browser tracks were visualized using the IGV (version 2.19.2).

### Statistical analysis

Statistical testing was done using GraphPad Prism version 10. Use tests are indicated in each figure legend. For co-transfer experiments, control and KO/OE cells were exposed to the same tumor or infection environment, thus paired Student’s *t* tests were used. One-way and two-way ANOVA were used to compare more than two groups. P value threshold was set at 0.05, with recommended corrections when multiple testing was performed. For all tests, *P < 0.05, **P < 0.01, and ***P < 0.001.

### Online supplemental material

Fig. S1 compares the *irf* family members’ expression level in TILs and chronic infection and shows gating strategies. Fig. S2 completes Fig. 4 with additional data for IRF8 level in the liver, additional ATAC-seq and ChIPseq data, and the effect of additional cytokines on IRF8 level. Fig. S3 shows a map of the vector used for KO and OE of IRF8, with the effect 48 h after activation on the indicated activation markers and completes Fig. 6, k–p, showing similar data in the LN. Fig. S4 shows the efficacy of the various KO and the strategy used for scRNA-seq. Fig. S5 complements Fig. 8 with additional GEO analysis. Table S1 shows the top 100 upregulated genes in IRF2, IRF4, and IRF8 KO versus control transduced TILs. Table S2 shows the top 100 downregulated genes in IRF2, IRF4, and IRF8 KO versus control transduced TILs. Table S3 shows antibodies used for flow cytometry and scRNA-seq multiplexing. Table S4 shows primer and sgRNA sequences.

## Supplementary Material

Table S1 shows the top 100 upregulated genes in IRF2, IRF4, and IRF8 KO versus control-transduced TILs.

Table S2 shows the top 100 downregulated genes in IRF2, IRF4, and IRF8 KO versus control-transduced TILs.

Table S3 shows antibodies used for flow cytometry and scRNA-seq multiplexing.

Table S4 shows primer and sgRNA sequences.

## Data Availability

All omics data are deposited on the GEO platform (GSE333230). All data are available from the corresponding author upon reasonable request.
