# Supplementary material for: The transcription factor IRF8 drives tumor-specific exhaustion in CD8+ T cells
Source: J Exp Med. 2026 Jun 22;223(8):e20252115. doi: 10.1084/jem.20252115 (PMC13285691; doi:10.1084/jem.20252115)
Supplement: Table S1 — shows the top 100 upregulated genes in IRF2, IRF4, and IRF8 KO versus control-transduced TILs. [file jem_20252115_tables1.pdf]

Upregulated in KO vs. Ctrl						
Common				Specific		
2, 4, 8	4, 8	2, 8	2, 4	8	4	2
Utf1	Ccl1	Ifit1	Bmyc	Penk	Dapl1	Pcsk1
Cdkn1a	Cnih2	Serpinb9b	Fabp5	Il4i1	Cd74	Slc6a19
Ms4a4c	Crtam	S100a11	Pla2g12a	Gzme	Eid2	Clic5
Dusp4	Xcl1	1700048O20Rik	Emb	Parp12	Irf2bp2	Fxyd7
Perp	Lta	Zbp1	As3mt	Gzmd	Sell	Klf4
Egr2	Gzma	Gucy1b1	Fam104a	Trbv15	Klf2	Esm1
Ifng	Tnf	Vat1	Rnft1	Ccl9	Arl5c	Prph
Rgs1	Slc17a6	Basp1	Fam53a	Rtp4	Ppp2cb	Arhgdig
Marcksl1	Rgcc	Gm44040	Dctn6	Ddx60	Tcf7	Tspan6
Mrps6	Id3	Cd70	Endod1	Tyrobp	Slamf6	Gm16341
Pacsin1	Tagap	Ier3	Bccip	Cxxc5	Tespa1	Nebl
Nr4a1	Slc9b2	Il1rl1	Tipin	Trav13-5	Hivep3	Gng3
Psme2	Itm2a	Itgax	Nop16	Eomes	Nme6	1110038F14Rik
Btg3	Cd81	D5Ertd579e			Trmo	Ccdc162
Ebna1bp2	Tnfsf14	Il1r2		Serpinb1b	Nsun4	Tmcc3
Hmgn1	Ephx1	Prf1		Gstt1	Bloc1s5	Lysmd2
Comtd1	Samd3	Gbp2		Ms4a4a	Zbtb32	Map2
Ass1	Cd9	Ccnd2		Serpine2	Atp23	Dnph1
	Nrn1	Mrpl55		Ube2l6	Camk2n1	Mt3
	Spry1	Tbc1d30		Plk2	Sult2b1	Spint1
	Pou2f2	Mak16		Zbtb39	Fastkd3	Gm36551
	Adssl1	Igfbp7		Ccdc184	Ado	Morn4
	Gm20400	Ly6a		Csf2	Dusp14	Gm26520
	Izumo1r	Tpmt		2010204K13Rik	Gramd4	Sorbs3
	Ggh	Gldc		Gm47162	Slc35f6	St3gal5
	Gadd45b	Tesc		Tmem63b	Med18	4930570N18Rik
	Pfdn2	Igtp		Tnfsf8	Tbc1d10a	Tanc2
	Gpm6b	Lad1		Ftl1-ps1	Gm20324	Jdp2
	Stard3	Isg15		Apobec2	Kcnn4	C1qtnf4
	Sema7a	Lilrb4b		Oas1a	Arl3	Hectd4
	Tnfsf9	Casp6		Slc25a42	Trmt61a	Npdc1
	Nfkbid	Fhl3		Hhex	Angptl4	Map6
	Smim36	Irgm1		Ppihl	Iigp1	Clec12a
	Arl4d	Ramp1		E430024P14Rik	Yars2	Osbpl8
	Gm10131	Psme2b		Gm49890	Casp4	Gbp5
	Pdcd1lg2	Herc6		Ifih1	Jagn1	Ckb
	Cd160	Tmbim4		Prkar2b	Zfp36	Acyp2
	Hsd17b10	Mlx		Klri2	Ninj1	Piwil2
	Ccl3	Gzmf		Sh2b3	Apex1	Ica1
	Gch1	Slco4a1		Gm43042	Ucp2	Misp3
	Ccl4	Gzmc		Etv4	Rad1	Gzmk
	Nrgn	Ptges3		Cdkn2a	Matk	Camk2n2
	Ctdnep1	Gnl1		Tmem106a	Evi2a	Stx2
	Plpp5	Pigf		Map3k8	Ube2q1	Klrh1
	Spp1	Cinp		Tubb2a	Asf1a	Rbm44
	Tnfrsf9	Isg20		Gm46620	Yrdc	Serpinf1
	Suox			F2rl2	H2-DMa	Lrp5
	Rpp25l			Shfl	Hacd2	Trim16
	Cenpv			Nr2f6	Zfpm1	Dscam
	Mrpl50			Gzmb	Slc29a1	Crip2
	Kpna2			Serpinb1a	Fancf	Camsap1
	Trappc4			Ms4a6d	Saysd1	Cd79b
	Pradc1			Cep85l	Mrpl39	Clstn1
	Bcl2a1b			Moap1	Ginm1	P4ha2
	Rgs16			Plxdc2	Lamp1	Pacs2
	Txnl4a			Mocs3	Ccdc71l	Prkcz
	Znhit3			Tmem120b	Prdx3	Trav8-2
	Timm17a			Ifi27	Entr1	Flywch2
	Vamp5			Insl6	0610009B22Rik	Dlg2
	Dpm2			Lilrb4a	Med21	Myl6b
	Surf2			Depdc1b	Gatd1	Spink2
	Ifi47			Eid2b	Rheb	Selenom
	Ccl5			Eno3	Nr4a3	Rhoh
	Fhl2			Dhx58	Polr2d	Numb
	Cnih4			Rhob	Pgm2	Tiprl
	Ap4s1			Dnajb5	Timm17b	Gm34794
	Sat1			Cd320	Klk8	Gbp3
	Dad1			Irf7	Sigmar1	Wnt10b
	Srgn			Gm49359	Rsl24d1	Jam2
	Orai1			Wsb2	Fos	Spats2
	Cib1			Cutc	Ndufaf7	Plcl1
	Jund			Pno1	Hexb	Cd274
	Tusc3			Mterf4	Cklf	Il12rb1
				Csf1	Aff3	Arhgef25
				Apmap	Cacybp	Phlda1
				Zfp69	Ppia	Castor1
				Lyrm4	Cenpb	Aplp1
				Tnfaip8l1	Nutf2	Gbp4
				Tgif1	Dnaja3	Acss2
				Zbtb8a	Atpsckmt	Pmm1
				Ifi208	Alkbh7	Lsr
				Rrm2	Creld2	Katna1
				Cdca5	Gtf2a2	Tnfrsf13b
				Rrp15	Cnep1r1	Dusp28
				Erh	Tvp23b	Coprs
				3110082I17Rik	Mrps17	Trim12c
				Eif4e	Sfxn3	Gbp7
				Polr2g	Atp6v0c	Snx8
				Tmem107	Pgap2	Gstt3
				Shcbp1	Eef1akmt2	Rab6b
				Mtap	3110040N11Rik	Mgst3
				Bst2	Slc25a20	Macir
				Bcat1	Cdk5	Hbegf
				Eola1	9130401M01Rik	Mt2
				Gadd45g	Srm	Meis3
				Hmgb3	Ostc	Tapbpl
				Fbxo5	Rnf7	Ehd3
				Zfpl1	Egln1	Zcwpw1
				Eif1b	Chchd4	Mt1
				Lsm2	Bcap31	Rab39b
				Asf1b	Lsm4	Arl4c

Table S1 | Top 100 upregulated genes in IRF2, IRF4, and IRF8 KO vs. control transduced TILs
