# Supplementary material for: The transcription factor IRF8 drives tumor-specific exhaustion in CD8+ T cells
Source: J Exp Med. 2026 Jun 22;223(8):e20252115. doi: 10.1084/jem.20252115 (PMC13285691; doi:10.1084/jem.20252115)
Supplement: Table S2 — shows the top 100 downregulated genes in IRF2, IRF4, and IRF8 KO versus control-transduced TILs. [file jem_20252115_tables2.pdf]

Downregulated in KO vs. Ctrl						
Common				Specific		
2, 4, 8	4, 8	2, 8	2, 4	8	4	2
Atp8a1	H2ac18	Tnfsf13b	Fcer1g	Klra5	H2ac10	Vwa5a
H4c14	H2ac13	Vmn2r96	Slfn9	Kcnq3	H2bc15	Zfp710
Maml2	Gm42743	3830403N18Rik	Cd244a	Sntb1	H3c11	Ube2l6
Zbtb20	H4c12	Gpnmb	C1qtnf6	Trpc1	H2ac20	Gm47015
Gm47283	H3c8	Aoah	H4c16	Maml3	H3c1	Apobec1
Heg1	H2bc3	Lsm5	Txk	Pnpla7	Myo10	Ctso
2900026A02Rik	H2ac15	Sidt1	Gm11131	Grk5	H2ac11	Ms4a6d
Dclk2	H2ac4	Slc15a2	Dleu2	Gm19705	H3c3	Armc3
Nop53	H3c6	Cd7	St8sia4	Prr5	H2bc18	Ctss
Chsy1	H2bc6	2610035D17Rik	Gm36723	Marveld2	Dpy19l4	B3gnt5
St6galnac3	H2ac6	Abi3	Gm20559	Cpq	Ctnnd2	Parp12
Arsb	H4c3	Art2b	Ptpre	Trgc2	H2ac22	P2ry14
Insyn2b	Gm10076	Myo3b	Phactr4	Arnt2	Pkp4	Gsdmd
Lysmd4	Mt2	Ifi211	5330438D12Rik	Atp11a	BC002059	Wls
mt-Nd3	Crmp1	Gm26827	Jarid2	Zfp931	Foxm1	Tmem63a
Zc3h7a	Snhg6	Lpp	Vps37b	Nckap1	H2bc14	Cyp4v3
Prkce	Rps21	Frmd4a	Tle4	Exoc3l	C030029H02Rik	Tmem106a
Trbv12-2	Rps18-ps4	Lrrk1	Sp4	Ppargc1b	Eif2ak4	Calhm6
Cemip2	Dscam	Styk1	Cep85	St6gal1	H2bc8	Mlkl
Arid1b	Exoc6b	Nfia	Lats1	C920021L13Rik	Ern1	Napsa
Phf21a	Slc9a1	Gm42031	Id2	Prrg1	AI987944	Usp18
Bcl2	Polr2l	Gm13571	Neurl3	Faxc	Gm19412	Oas1a
Tox	Mt1	Rreb1	Chn2	Neil2	Cep128	Dhx58
H4c4	Atrn	Gngt2	Wdr95	B3gat2	9430015G10Rik	Gnb4
mt-Co3	Rpl38	Gns	Fbxl17	5430403G16Rik	Rttn	Naip2
H2ac24	Ccnb1ip1	Lamp2	Rlf	Fbxo32	H1f2	Mtm1
Mbd5	Atp5k	Gm44175	Cwf19l2	Uvssa	Ppp1r12b	Trav15-2-dv6-2
Dennd1a	Tubgcp6	Ncf1	Jmjd1c	Tmtc2	Ccp110	Phf11a
Lyn	Bola2	Cdk8	Samd9l	Inpp5a	Spout1	H2-DMa
Setbp1	Med13l	Ncoa1	Pik3cd	Heph	Itpr3	Rgs12
Peak1	Ptpn12	4930444A19Rik	Ccr5	Optn	Vps13c	Parp3
Bicra	Phlpp1	Rere	Mef2a	Zfp984	Adcy3	Abcb1a
Gm20628	Slirp	Ssbp2	Ifi203	Tarsl2	Fbxo33	Il1rap
Tmem258	Rpl37	Cdkl3	Parp14	Fam20a	Cip2a	Gm44148
Sytl2	Rad51b	D130009I18Rik	H2bc9	Zfp709	Atxn1l	Ddx60
AY036118	Tomm7	Filip1l	Wbp1l	Ajuba	Mrtfb	Psrc1
H4c8	Rpl39	Tsix	Tab2	Tnk2	Chst10	Slc6a13
Itpkb	Rpl41	Gm32036	Crebrf	Gm49797	Map3k3	Insl6
Zfp407	Nbeal2	Purb	H2bc11	Gm17106	Slc23a2	Igsf5
Tcf20	Rps28	Tcf4	Pik3ap1	Zhx3	Lss	Sp140
Fbxo11	Ndufa3	Gm49417		Zfp780b	Pcnx3	Tmem171
Gm44174	Mmp16	6530409C15Rik		Firre	Il1rapl1	Hipk2
Thada	Anapc13	Baiap3		Cul9	Gga3	Sp110
Mbnl1	Ppard	Ankrd40		Enox2	Ankib1	Adgrg5
Dgkh	Rps27rt	Dcaf5		Eef2k	Zbtb11	Tmem50b
Ust	Norad	Sars		1700028E10Rik	Tmed8	Ccr8
Runx3	Lst1	Zfp831		Castor2	E2f7	Khk
Cdyl	Pet100	Naaladl2		Tsga10	Scd2	Mamdc2
Rps29	Kmt2d	Crim1		Zbtb37	Nkain2	Klra3
H3c7	Rpl37a	Rps6ka5		Pld2	Spc25	Shfl
Picalm	Ski	Scpep1		H2bc12	Rad54b	Trappc2l
Ptprj	Airn	Mid1		Slc12a7	Def8	Rnf213
mt-Atp8	Myo5a	Rps6ka3		Adrb1	Arrb1	Fggy
Gm17494	Tmsb10b	Slc10a7		Disp1	Pbx3	9930111J21Rik1
Slc9a9	Med12l	Znrf3		Map2k6	Ccr2	Trp53inp2
Eif4a2	Sipa1l1	Ndufaf8		E130307A14Rik	Ankra2	Zfp971
Fryl	Trim30d	Nf1		Stk3	Cbx6	Ifitm3
Arhgap15	Ndufb1	Chchd5		A330023F24Rik	Atr	Oas3
Arid1a	Ppwd1	Snx13		Sgms1	B3gntl1	Csf1
Mlxip	Lockd	Lncpint		Pip5k1b	Akt3	Gm28379
Cdc14a	Wipf2	Myo18a		AU020206	Ndufv3	Cyria
Rps27	Gm39323	Gm36975		Tecpr2	Ndufa2	Tor3a
Prkacb	Drosha	Impact		Arhgap39	Fnip1	Ly75
Pvt1	Gm36738	Polr2a		Rbm48	Apobr	Trim26
Chst11	Rpl36	Ehbp1l1		Pag1	Tent5a	Tmem9
Zcchc7	Romo1	Abcc4		Dph6	Arid3b	Gm11867
Srgap3	Dock9	Hmbox1		Slc20a2	Pou2f1	Hsd17b11
Aak1	Rrbp1	Slc16a10		Arhgap35	Cdin1	Ctns
Inpp5d	Neat1	Rfx3		Rapgef2	Uqcr11	Scly
Setd5	Rps15	Gm37240		Slc38a9	Rad21	Tnfsf8
Zfp512	mt-Cytb	Klrc2		Pls1	Mki67	Snx10
Snhg8	Dpm3	Hdac4		Plekhg3	H1f3	Pltp
Prrc2b	Nmt2	L3mbtl3		Tango6	Nr2c2	Plscr4
Sik3	Rpl36a	Chka		Bbs9	Lin52	Cnbd2
Zeb1	Clasp1	Zfp652		Ank	Hjurp	Mr1
	Tmem256	Btaf1		Lrrc4	Srbd1	Gm14636
	Rps12	Dusp6		Sipa1l3	Ktn1	Rasgef1b
	Adam19	Crebbp		Usf2	Heatr5b	Prr36
	Cox7c	Plcl2		Pkd1	Epb41	Tmem79
	Rpl13a	Ptbp2		Msi2	Spty2d1	4933412E12Rik
	Unkl	Gfod1		Il12rb1	Tbc1d5	Parp8
	Pacs1	Cdk19		Tmcc3	Ppp1r16b	Grn
	Hectd4	Lcorl		Ikzf5	Mt3	Cd74
	Mctp2	Pcnx		Iqgap2	Trdv5	Rtp4
	Ralgapb	Xpr1		Tnfaip1	E2f2	Ogfrl1
	Xist	Itpr2		Dcun1d3	Rplp2	Pvrig
	Dock8	Nol6		Usp20	S100a4	A330040F15Rik
	Gse1	Fcho2		Ext1	Crip1	Gm17655
	Map3k14	Tnrc6a		Nedd4	Mink1	Nod1
	Abca2	Mtmr3		Lcn4	Usp16	Bcl6
	Son	Psme4		Oprm1	Ncapg2	Rasa4
	Eif2ak3	Cul3		Dcaf6	Ganab	Socs5
	Atg2a	Gm15283		Agbl5	Zfp639	Cdkn2a
	Ssh2	Bcas3		Clcn5	Cops9	Mov10
	Prkch	Immp2l		Zscan22	Znfx1	Rps18-ps6
	Upf2	Sesn1		Ireb2	Rplp1	Hopxos
	Cdk6	Nlrc3		Kat14	Mrpl52	Trav13-4-dv7
	Trps1	Sos2		Efcab11	Cenpw	2900041M22Rik
	S100a6	Usp15		Marchf6	Dzip3	Dusp7
	Mir142hg	Hipk1		Pik3c2a	Abl2	Ccrl2
	Rpl34	Abtb2		Slc30a4	Rexo5	Ppp2cb

Table 2 | Top 100 downregulated genes in IRF2, IRF4, and IRF8 KO vs. control transduced TILs
