# Supplementary material for: The transcription factor IRF8 drives tumor-specific exhaustion in CD8+ T cells
Source: J Exp Med. 2026 Jun 22;223(8):e20252115. doi: 10.1084/jem.20252115 (PMC13285691; doi:10.1084/jem.20252115)
Supplement: Table S3 — shows antibodies used for flow cytometry and scRNA-seq multiplexing. [file jem_20252115_tables3.pdf]

Application	Target	Flurochrome	Dilution	Clone	Cat.#	Source
Flow Cytometry	CD3	PerCP-Cy5.5	100	145-2C11	100328	BioLegend
	CD8	BUV395	100	53.6.7	363-0081-82	Invitrogen
		APC-Cy7	200	53.6.7	100714	BioLegend
		FITC	200	53.6.7	-	DO@Unil
		Biotin	100	53.6.7	-	DO@Unil
	CD44	BUV737	200	IM.781	367-0441-82	Invitrogen
	CD45.1	BV650	200	A20.1	416-0453-82	Invitrogen
		BUV737	100	A20.1	367-0453-82	Invitrogen
	CD45.2	PerCP-Cy5.5	100	104	109828	BioLegend
		BUV805	100	104	368-0454-82	Invitrogen
		BUV395	100	104	363-0454-82	Invitrogen
	CD90.1	eFluor450	100	H1S51	48-0900-82	Invitrogen
		APC	100	H1S51	17-0900-82	Invitrogen
	Tim-3	BV421	100	RMT3-23	119723	BioLegend
		BV785	100	RMT3-23	119725	BioLegend
	PD1	PE-Cy7	100	29F.1A12	135216	BioLegend
		BV711	100	29F.1A12	135231	BioLegend
		eFluor450	100	RMPI-30	48-9981-82	Invitrogen
	LAG3	PE-Dazzle594	200	C9B7W	125224	BioLegend
	TOX	eFluor660	200	TXRX10	50-6502-82	Invitrogen
		PE	200	TXRX10	12-6502-82	Invitrogen
	IRF2	-	500	EPR4644(2)	ab124744	abcam
	IRF4	Pacific Blue	200	IRF4.3E4	646418	BioLegend
		PE-Cy7	200	IRF4.3E4	646413	BioLegend
	IRF7	PE	200	MNGPKL	12-5829-82	Invitrogen
	IRF8	PE	200	V3GYWCH	12-9852-82	Invitrogen
		APC	200	V3GYWCH	17-9852-82	Invitrogen
	IFNγ	PE-Cy7	100	XMG1.2	505826	BioLegend
		PerCP-Cy5.5	300	XMG1.2	505821	BioLegend
	GzmB	FITC	200	GB11	515403	BioLegend
		PE-Dazzle594	200	QA16A02	372216	BioLegend
	TNFα	Pacific Blue	100	MP6-XT22	506318	BioLegend
	IL-2	APC	100	JES6-5H4	503810	BioLegend
	IFNAR1	APC	100	MAR1-5A3	127313	BioLegend
	αRabbit IgG	FITC	200	Polyclonal	11-4839-81	Invitrogen
	Ly108 (Slamf6)	Biotin	200	13G3-19D	13-1508-82	Invitrogen
	LIVE/DEAD Fixable Aqua Dead cell Stain Kit	BV510	500	-	L34957	Invitrogen
	Zombie NIR (Fixable Viability kit)	NIR	1000	-	423106	BioLegend
	CD3	AmCyan	100	SK7	339186	BD Biosciences
	CD4	Biotin	100	SK3	347321	BD Biosciences
	CD8	BV786	100	RPA-T8	563823	BD Biosciences
	CD45RA	PE-Cy5	100	HI100	555490	BD Pharmingen
	CCR7	BV421	100	G043H7	353208	BioLegend
	PD-1	PerCP-eFluor710	100	eBioJ105	46-2799-42	eBiosciences
	TIM3	PE-Cy7	100	F38-2E2	25-3109-42	Invitrogen
	LAG3	FITC	100	17B4	ALX-804-806F-C100	Enzo
	Biotin	BV605	100	-	405229	BioLegend
	Biotin	BV650	200	-	405232	BioLegend
	Biotin	APC-Cy7	100	-	405208	BioLegend
Hashtagging scRNA-seq multiplexing	TotalSeq™-C0956	APC-Streptavidin	400	-	405283	BioLegend
	TotalSeq™-C0957	APC-Streptavidin	400	-	405285	BioLegend
	TotalSeq™-C0960	APC-Streptavidin	400	-	405157	BioLegend
	TotalSeq™-C0976	APC-Streptavidin	400	-	405195	BioLegend
	TotalSeq™-C0977	APC-Streptavidin	400	-	405197	BioLegend
	TotalSeq™-C0978	APC-Streptavidin	400	-	405199	BioLegend
	TotalSeq™-C0979	APC-Streptavidin	400	-	405337	BioLegend
	TotalSeq™-C0980	APC-Streptavidin	400	-	405339	BioLegend

Table S3 | Antibodies used for flow cytometry and scRNA-seq multiplexing
