# Supplementary material for: The transcription factor IRF8 drives tumor-specific exhaustion in CD8+ T cells
Source: J Exp Med. 2026 Jun 22;223(8):e20252115. doi: 10.1084/jem.20252115 (PMC13285691; doi:10.1084/jem.20252115)
Supplement: Table S4 — shows primer and sgRNA sequences. [file jem_20252115_tables4.pdf]

Application	Name	Sequence
qPCR	Primer IRF2 For	CTAACAACACTCACACCTTGCG
	Primer IRF2 Rev	TTCCTGGTTGATGCTTTCCTGT
	Primer IRF4 For	GAATTGGTCGAGAGGAGCCA
	Primer IRF4 Rev	AGTTATGAACCTGCTGGGCT
	Primer IRF8 For	GAGCGAAGTTCCTGAGATGG
	Primer IRF8 Rev	TGGGCTCCTCTTGGTCATAC
	Primer B2M For	AGACTGATACATACGCCTGCAG
	Primer B2M Rev	GCAGGTTCAAATGAATCTTCAG
CRISPR/Cas9 KO	sgRNA IRF2	TTGCTAGGAAAGCATCAACC
	sgRNA IRF4	CCTGTGACGTTTGGCCCACG
	sgRNA IRF8	TCGACAGCAGCATGTACCCG
	sgRNA IFNAR1	TCAGTTACACCATACGAATC
	sgRNA Control	AAAAAGTCCGCGATTACGTC

Table S4 | Primer and sgRNA sequences
